# Arabidopsis Plastidial Folylpolyglutamate Synthetase Is Required for Seed Reserve Accumulation and Seedling Establishment in Darkness

**DOI:** 10.1371/journal.pone.0101905

**Published:** 2014-07-07

**Authors:** Hongyan Meng, Ling Jiang, Bosi Xu, Wenzhu Guo, Jinglai Li, Xiuqing Zhu, Xiaoquan Qi, Lixin Duan, Xianbin Meng, Yunliu Fan, Chunyi Zhang

**Affiliations:** 1 Biotechnology Research Institute, Chinese Academy of Agricultural Sciences, Beijing, People’s Republic of China; 2 National Key Facility for Crop Gene Resources and Genetic Improvement (NFCRI), Beijing, People’s Republic of China; 3 Huazhong Agricultural University, Wuhan, People’s Republic of China; 4 Beijing Institute of Pharmacology and Toxicology, Beijing, People’s Republic of China; 5 Institute of Botany, Chinese Academy of Sciences, Beijing, People’s Republic of China; Iowa State University, United States of America

## Abstract

Interactions among metabolic pathways are important in plant biology. At present, not much is known about how folate metabolism affects other metabolic pathways in plants. Here we report a T-DNA insertion mutant (*atdfb-3*) of the plastidial folylpolyglutamate synthetase gene (*AtDFB*) was defective in seed reserves and skotomorphogenesis. Lower carbon (C) and higher nitrogen (N) content in the mutant seeds than that of the wild type were indicative of an altered C and N partitioning capacity. Higher levels of organic acids and sugars were detected in the mutant seeds compared with the wild type. Further analysis revealed that *atdfb-3* seeds contained less total amino acids and individual Asn and Glu as well as NO_3_^−^. These results indicate significant changes in seed storage in the mutant. Defects in hypocotyl elongation were observed in *atdfb-3* in darkness under sufficient NO_3_^−^ conditions, and further enhanced under NO_3_^−^ limited conditions. The strong expression of *AtDFB* in cotyledons and hypocotyl during early developmental stage was consistent with the mutant sensitivity to limited NO_3_^−^ during a narrow developmental window. Exogenous 5-formyl-tetrahydrofolate completely restored the hypocotyl length in *atdfb-3* seedlings with NO_3_^−^ as the sole N source. Further study demonstrated that folate profiling and N metabolism were perturbed in *atdfb-3* etiolated seedlings. The activity of enzymes involved in N reduction and assimilation was altered in *atdfb-3*. Taken together, these results indicate that AtDFB is required for seed reserves, hypocotyl elongation and N metabolism in darkness, providing novel insights into potential associations of folate metabolism with seed reserve accumulation, N metabolism and hypocotyl development in Arabidopsis.

## Introduction

The role of seeds is to propagate offspring. In *Arabidopsis thaliana*, seed development can be divided into three stages: cell division or the pre-storage phase, maturation or the storage phase, and the desiccation phase [1], [2]. Large quantities of carbon (C) and nitrogen (N) are stored in maturing seeds, mainly in the form of large insoluble compounds [3]. The major storage compounds that accumulate in mature seeds are triacylglycerols (TAGs) and seed storage proteins (SSPs), accounting for 30–45% of the seed dry weight. Small amounts of carbohydrate in the form of sucrose are stored within cotyledons [1], [3], [4], [5], [6]. SSPs, including soluble proteins and non-soluble proteins, include two predominant classes, namely, 12S globulin and 2S albumin [1], [4], [7]. Seed storage accumulation is regulated by many factors, such as hormones, sugars, master regulator genes and transcriptional factors [1]. These seed reserves are used to fuel germination and post-germinative seedling establishment until seedling photosynthesis autotrophy can be efficiently established [4].

Seed germination and post-germinative seedling establishment are metabolically distinct [8], [9]. Germination initiates with release from dormancy and seed imbibition and is completed when the radicle emerges through the seed coat [10]. At the beginning of germination, seed reserves other than lipids (TAG) are rapidly converted to soluble metabolites (e.g. glycolysis products, organic acids, and amino acids) that can be transported throughout the seedling to support growth, while the breakdown of seed oil storage TAG is used for subsequent seedling establishment after the radicle has emerged [3], [8], [9], [11], [12], [13]. Following germination, TAG is broken down to yield free fatty acids (FAs) and glycerol, both of which are ultimately converted to sugars required for post-germinative seedling development [6], [12]. The *sdp1* mutant, containing a mutation in sugardependent1 (SDP1), which encodes a patatin domain TAG lipase that initiates TAG breakdown in germinating seeds, displayed slightly delayed seed germination and a much slower post-germinative growth rate than the wild type [14]. Seedlings grown in darkness showed skotomorphogenesis, which is characterized by elongated weak hypocotyls, closed cotyledons, and shortened roots [15]. Seedling establishment and hypocotyl elongation are driven by the catabolism of TAG under dark conditions. Mutants (*icl* and *pck1*) defective in TAG mobilization show shortened hypocotyls in darkness, but hypocotyls could be rescued by providing alternative C sources, such as sucrose [16], [17]. N metabolism is also essential for hypocotyl growth. In conifer plants grown in the dark, a portion of N mobilized from the megagametophyte is diverted toward the hypocotyl shortly after germination to produce high levels of Asn, which serves as a reservoir of N to meet subsequent specific developmental demands [18].

Tetrahydrofolate (THF) and its derivatives are collectively called folates. Most cellular folates carry a short poly-γ-Glu tail, which is believed to affect their efficacy and stability. The tail can be removed by γ-glutamyl hydrolase (GGH), a vacuolar enzyme who has an important influence on polyglutamyl tail length and hence on folate stability and cellular folate content [19]. Folylpolyglutamate derivatives are central cofactors for many folate-dependent enzymes [20], [21], [22], [23], [24], [25]. During the germination process, *de novo* synthesis of THF occurs in pea (*Pisum sativum*) cotyledons, and the inhibition of THF *de novo* synthesis using folate analogs blocks seedling development [26], [27], [28]. The cotyledonary folate pool contains principally methylated derivatives [28]; the concentration of folylpoly-Glu derivatives increases gradually during germination [29], and the accumulation of folates peaks 3 days after sowing [27].

Plants with defective folate biosynthesis and metabolism showed various aberrant seed and seedling phenotypes. For example, the *globular arrest1* (*gla1*) mutant, which contains a mutation in dihydrofolate synthetase folylpolyglutamate synthetase (DHFS-FPGS) homolog A (DFA), encoding a functional mitochondrial matrix-localized dihydrofolate (DHF) synthetase, exhibited defective embryonic development and did not undergo transition to the heart stage [23], [30]. The double knockout (dKO) mutation of 10-formyl-THF deformylase genes, At4g17360 and At5g47435, resulted in defective embryo development, with cells arresting between the heart and early bent cotyledon stages. Mature seeds of dKO were shriveled, accumulated low amounts of lipids, and failed to germinate [31]. A mutation in *AtDFB*, which encodes the plastidial folylpolyglutamate synthetase (FPGS) isoform, displayed short primary roots with a disorganized quiescent center [24], [32]. A mutation in *AtDFC*, which encodes the mitochondrial FPGS, was characterized based on its altered N metabolism and enhanced phenotypes to low N stress, providing novel insights into folate biosynthesis and N utilization during early seedling development [33]. To date, the role of folate during skotomorphogenesis in plants remains poorly understood.

In this report, a mutant (*atdfb-3*) carrying a T-DNA insertion in the *AtDFB* gene was characterized for its altered seed reserves and defective seedling establishment with shortened hypocotyls under dark conditions. Early post-germinative growth (before 3 days) in *atdfb-3* required external NO_3_^−^ sufficient conditions, and exogenous application of 5-formyl-tetrahydrofolate (5-F-THF) restored hypocotyl length in *atdfb-3* when NO_3_^−^ was as the sole N source in the medium. The defective hypocotyl elongation could be due to altered seed storage, perturbed folate and N metabolism in *atdfb-3*. This report provides novel insights into a potential associations of folate metabolism with seed reserve accumulation, N metabolism and hypocotyl development elongation in darkness in Arabidopsis.

## Results

### Reduced seed size and altered C/N partition capacity in mature seeds of *atdfb-3*

A previous report demonstrated that the vegetative phenotype of *atdfb* (*fpgs1*, SALK_133817) did not differ visually from the wild type under light conditions [34]. In this report, SALK_015472 with a T-DNA insertion in the sixth intron of *At5g05980* (Figure S1A in File S1), which encodes the plastidial isoform of FPGS (AtDFB), was obtained from the Arabidopsis Biological Resource Center (The Ohio State University) and named *atdfb-3,* as described previously [32].

**Figure 1:**
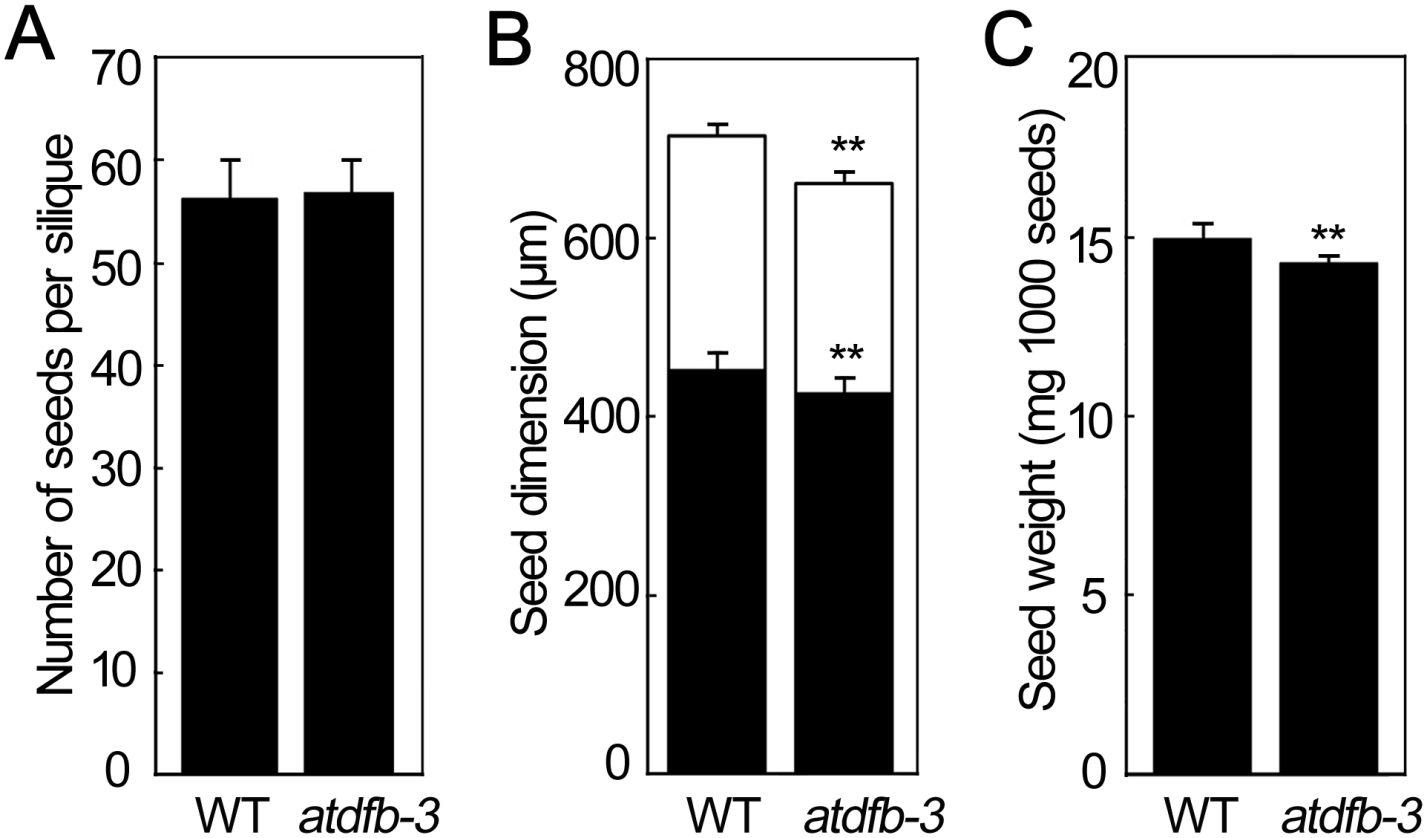
Seed characteristics in WT and *atdfb-3*. (A) Number of seeds per silique. (B) Seed length (black bars) and width (white bars). (C) Seed weight. Data represent means ± SD. A, n = 30; B, n = 3, and each replicate contained 30 seeds. Seeds were viewed using a ZEISS Imager M1 DIC microscope and measured using ImageJ; C, n = 5, and each replicate consisted of a pool from 10 plants. Bars with ** indicate a highly significant difference at *P*<0.01 (Student’s *t*-test).

First, characteristics of seeds harvested from *atdfb-3* and wild-type plants grown under light conditions, such as seed number per silique and 1000 seed weight, were examined. No significant difference was observed in seed number per silique between *atdfb-3* and the wild type (Figure 1A); however, the width and length of mature *atdfb-3* seeds were slightly but significantly smaller than those of the wild type (Figure 1B). The reduction in dimensions was somewhat reflected by the seed weight, with a significant decrease of 5% in *atdfb-3* compared with the wild type (Figure 1C).

**Figure 2:**
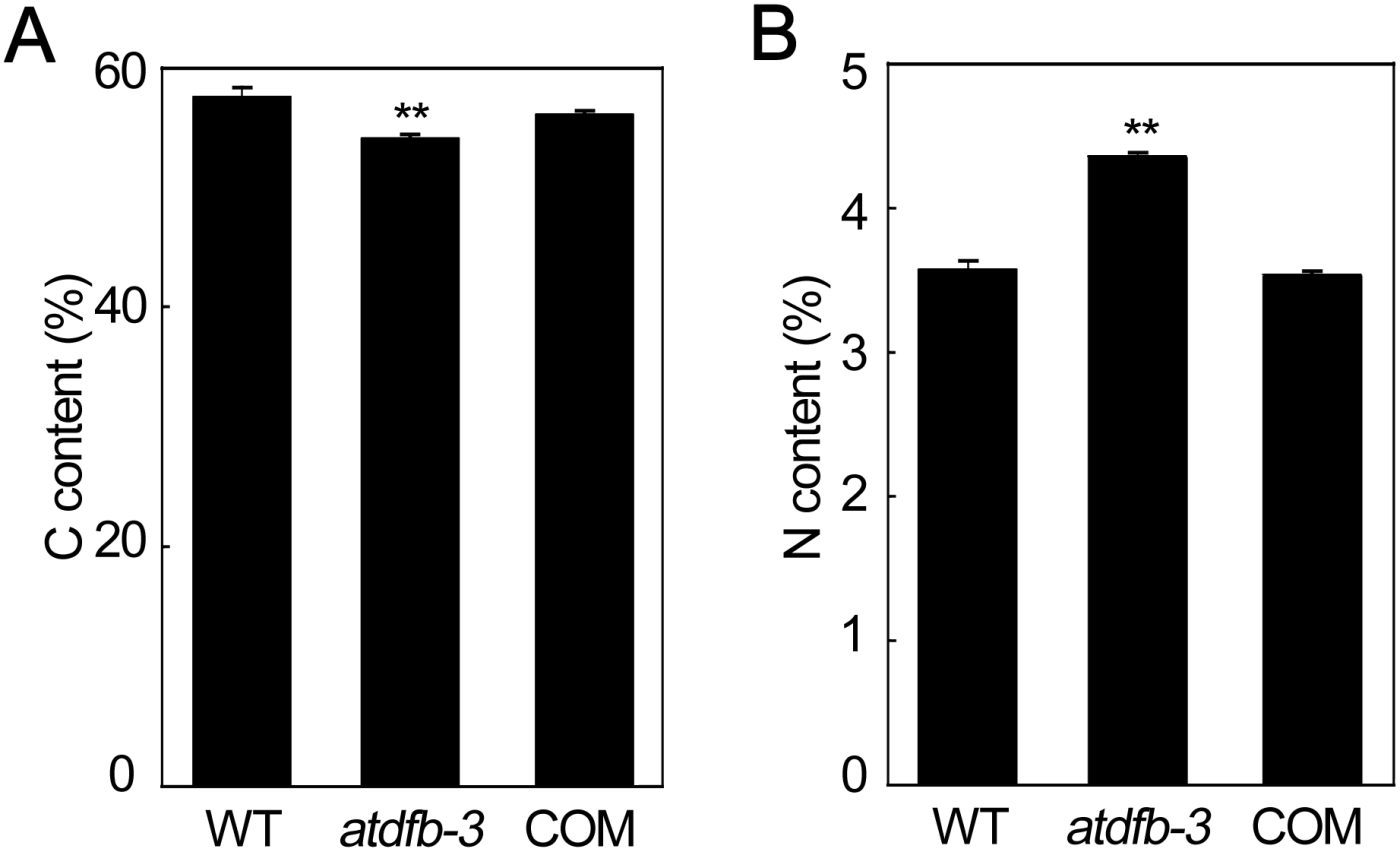
C and N contents in mature WT, *atdfb-3* and *AtDFB* complemented (COM) seeds. (A) C content. (B) N content. Data represent means ± SD. n = 4, and each replicate consisted of 10 mg DW of pooled plant material. Bars with ** indicate a highly significant difference at *P*<0.01 (Student’s *t*-test).

Next, we explored seed reserves in mature *atdfb-3* seeds. We found that C and N levels in *atdfb-3* seeds were 94% and 122%, respectively, compared with wild-type levels (Figure 2A and B). In *AtDFB* complemented plants (Figure S1B and C in File S1), these changes were restored to wild-type levels (Figure 2A and B), indicating they were due to the loss of function of *AtDFB*. These results indicated the altered C and N partitioning capacity observed in *atdfb-3* was due to the loss of function of *AtDFB*.

### Altered C and N metabolites in *atdfb-3* seeds

**Figure 3:**
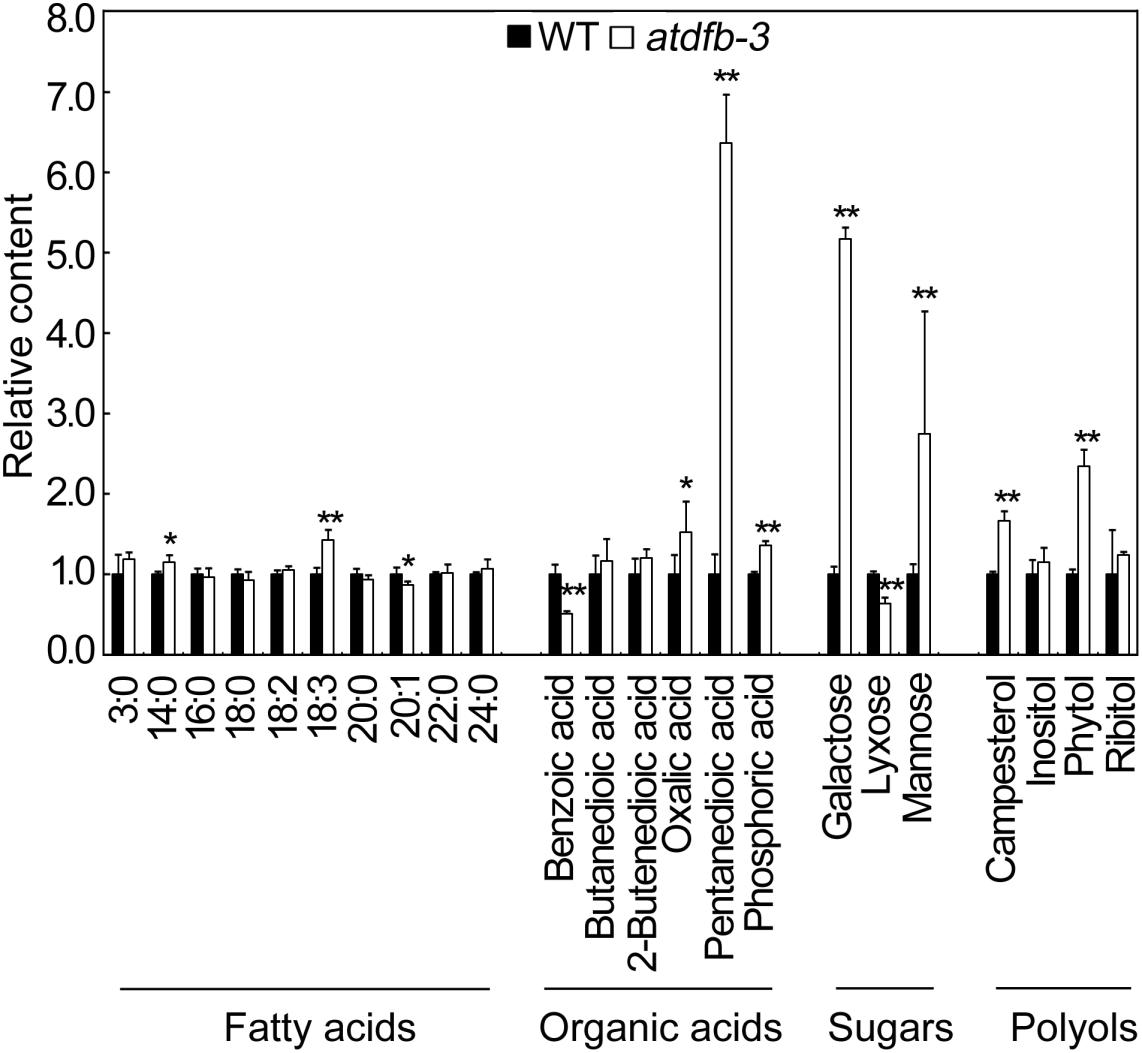
Metabolite profiles in WT and *atdfb-3* seeds. Propanoic acid (3∶0), tetradecanoic acid (14∶0), hexadecanoic acid (16∶0), octadecanoic acid (18∶0), 9,12-octadecadienoic acid (18∶2), linolenic acid (18∶3), eicosanoic acid (20∶0), 11-eicosenoic acid (20∶1), docosanoic acid (22∶0), and tetracosanoic acid (24∶0). n = 5, and each replicate consisted of 20 mg of pooled seeds. Data represent means ± SD. White bars with * indicate a significant difference at *P*<0.05, and ** indicates a highly significant difference at *P*<0.01 (Student’s *t*-test).

We analyzed metabolites in *atdfb-3* and wild-type seeds using gas chromatography time-of-flight mass spectrometry (GC-TOF-MS). A total of nine metabolites, including two FAs (14∶0 and 18∶3), three organic acids (oxalic acid, pentanedioic acid, and phosphoric acid), two sugars (galactose and mannose), and two polyols (campesterol and phytol) were higher and three metabolites (20∶1, benzoic acid, and lyxose) were lower in *atdfb-3* than in the wild type (Figure 3). In addition, the contents of other metabolites (mainly fatty acids) detected in *atdfb-3* were similar to those in the wild type. These results suggested that the mutation in *AtDFB* altered C-rich metabolites accumulation in mature seeds.

**Figure 4:**
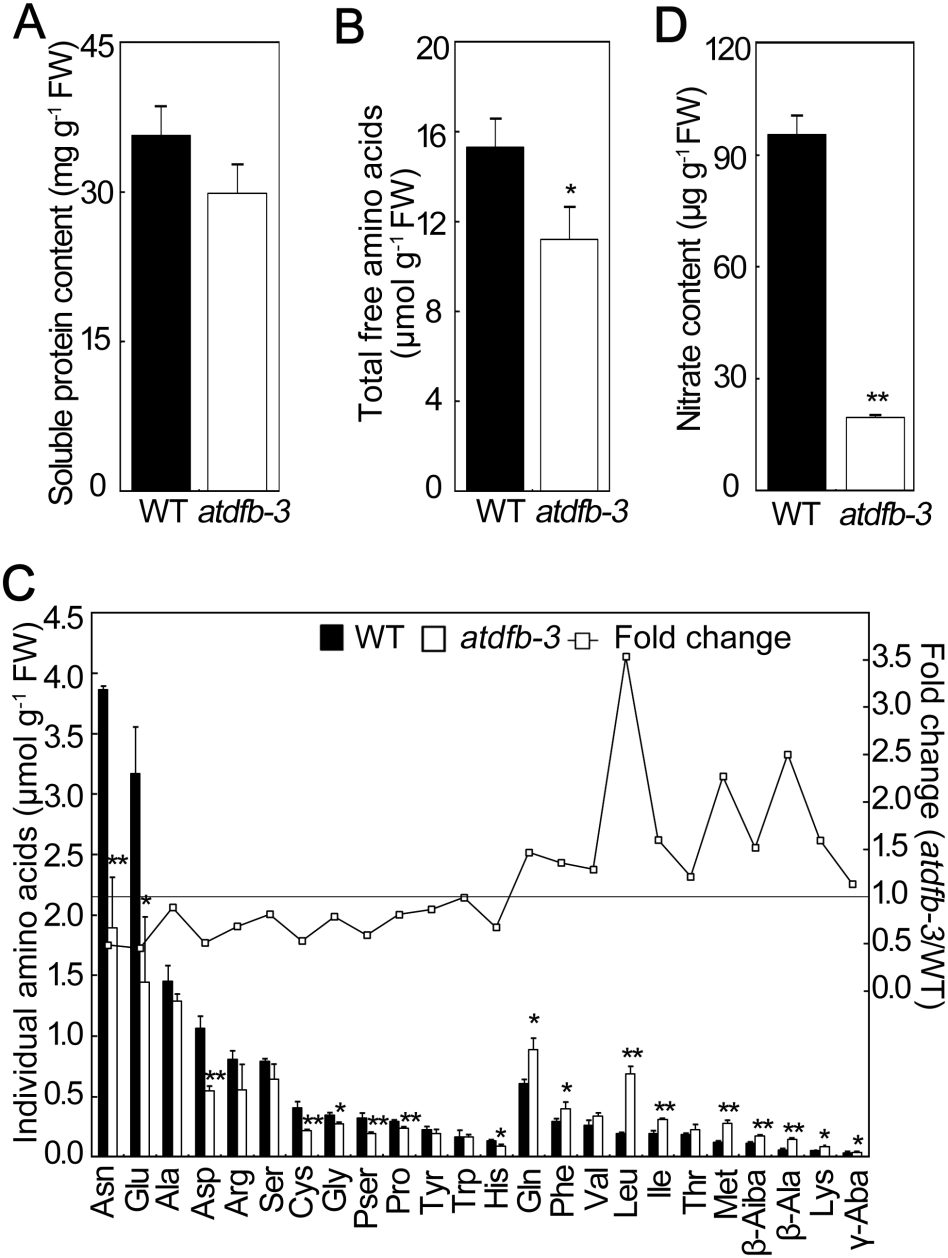
Contents of N-rich metabolites in WT and *atdfb-3* seeds. (A) Soluble protein. (B) Total amino acids. (C) Individual amino acids. (D) NO_3_^−^ content. Data represent means ± SD. n = 3. A, each replicate consisted of 50 mg pooled plant material; B and C, each replicate consisted of 300 mg pooled plant material; D, each replicate consisted of 1 g pooled plant material. Bars with * indicate a significant difference at *P*<0.05, and ** indicates a highly significant difference at *P*<0.01 (Student’s *t*-test).

The level of soluble protein was not significantly different from the wild type in *atdfb-3* (Figure 4A), while the total free amino acids were significantly less in *atdfb-3* seeds than in the wild type (Figure 4B). Many individual amino acids accumulated to lower levels in *atdfb-3* than in the wild type, such as Asn, Glu, Asp, Cys, Gly, Pser, Pro, and His. Asn and Glu were both 50% less than the wild type, accounting for the main shortage of total amino acids in *atdfb-3* (Figure 4C). In contrast, some other amino acids accumulated more in *atdfb-3* than in the wild type, such as Gln, Phe, Leu, Ile, Met, β-Aiba, β-Ala, Lys, and γ-Aba. Among them, Leu in *atdfb-3* accumulated to the highest level: 2.5-fold higher than that of the wild type (Figure 4C). As a result, total amino acids in *atdfb-3* seeds were 27% lower than in the wild type, and the Gln/Glu ratio in *atdfb-3* (0.62) was higher than that of the wild type (0.19). Additionally, the NO_3_^−^ content in *atdfb-3* seeds was only 21% of that in the wild type (19.6 µg g^−1^ in *atdfb-3* vs 95.4 µg g^−1^ in the wild type; Figure 4D). These results indicated that the mutation in *AtDFB* reduced the accumulation of N-rich metabolites.

### Sufficient N supply (but not C) was required for early post-germinative growth of *atdfb-3* in darkness

**Figure 5:**
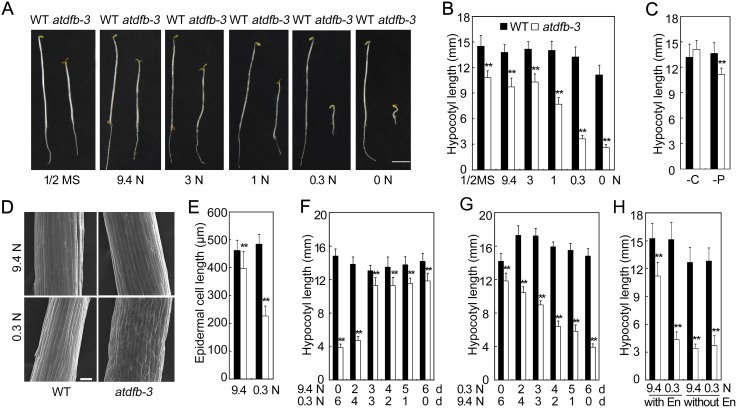
Maladjustment time window in *atdfb-3* etiolated seedlings. (A) Images of 6-day-old etiolated WT and *atdfb-3* seedlings grown on different amounts of nitrogen (N). Scale bar, 3 mm. (B) Hypocotyl lengths of 6-day-old etiolated WT and *atdfb-3* seedlings grown on different amounts of nitrogen (N). (C) Effects of carbon (C)-free or phosphate (P)-free media on the hypocotyl lengths of 6-day-old WT and *atdfb-3* etiolated seedlings. (D) FE-SEM image of hypocotyl cells of 6-day-old etiolated WT and *atdfb*-3 seedlings grown on 9.4 N and 0.3 N medium. Scale bar, 100 µm. (E) Hypocotyl cell length in 6-day-old etiolated WT and *atdfb*-3 seedlings on 9.4 N and 0.3 N medium. (F) Hypocotyl length of 6-day-old WT and *atdfb-3* etiolated seedlings. Both genotypes seedlings were grown on 9.4 N medium for 0 to 6 days and then transferred to 0.3 N medium for the remaining days (0 to 6 days). (G) Hypocotyl length of 6-day-old WT and *atdfb-3* etiolated seedlings. Seedlings of both genotypes were grown on 0.3 N medium for 0 to 6 days and then transferred onto 9.4 N medium for the remaining days (0 to 6 days). (H) Hypocotyl length of 6-day-old WT and *atdfb-3* etiolated seedlings with or without endosperm in 9.4 N and 0.3 N media. Data represent the means ± SD (in panel B, C, E, F, G and H, n = 15). White bars with * indicate a significant difference at *P*<0.05, and ** indicates a highly significant difference at *P*<0.01 (Student’s *t*-test).

The post-germinative growth of the mutant was investigated in the dark under various N conditions. After growing on half-strength MS medium (30 N) for 6 days, *atdfb-3* had shortened hypocotyls and primary roots as well as expanded cotyledons and a larger apical hook curvature than the wild type (Figure 5A and B). Similar results were obtained when ammonium (NH_4_^+^) was omitted and 9.4 mM or 3 mM NO_3_^−^ (9.4 N or 3 N, respectively) was added to the medium (Figure 5A). Interestingly, when the amount of NO_3_^−^ in the medium was decreased further (less than 3 mM), there were no significant changes in the lengths of hypocotyls of the wild-type seedlings, but the mutant displayed even shorter hypocotyls (Figure 5A and B). When the medium was supplemented with 0.3 mM NO_3_^−^ (0.3 N) or 0 N, these hypocotyl and primary root phenotypes of *atdfb-3* differed significantly from those of the wild type. The cotyledons of *atdfb-3* were folded similarly to those of the wild type; however, the apical hook curvature in *atdfb-3* appeared larger than that in the wild type (Figure 5A). Next, we explored the hypocotyl phenotype further. Interestingly, when NH_4_^+^ was used as the sole N source in the medium, it could not be used for hypocotyl development in *atdfb-3* (unlike NO_3_^−^), whether at higher (9.4 and 3 mM) or lower (1 and 0.3 mM) concentrations (Figure S2A and B in File S1). In addition, the mutant could not utilize organic nitrogen Asn or Gln in the medium under dark conditions (Figure S2C in File S1). These results indicated that *atdfb-3* was sensitive to external NO_3_^−^ concentrations during skotomorphogenesis, and the hypocotyl elongation in *atdfb-3* required an external, sufficient NO_3_^−^ supply.

Mutant and wild-type seedlings were also grown on media with other nutrient deficiencies. When grown on C-free medium, there was no obvious difference in hypocotyl length between *atdfb-3* and the wild type (Figure 5C). On phosphate (P)-free medium in darkness, the hypocotyl length of *atdfb-3* was 80% compared with that of the wild type (Figure 5C); the ratio was similar to that under N-sufficient conditions (3 mM or higher NO_3_^−^ concentration). These results indicated *atdfb-3* had a specific response to the external NO_3_^−^ supply (but not C or P) during seedling establishment in darkness.

Since hypocotyl elongation in *atdfb-3* was significantly inhibited by 0.3 N (0.3 mM NO_3_^−^), and the phenotype of the mutant on 9.4 N was similar to that on 1/2 MS, 0.3 N and 9.4 N were used as N-limited and N-sufficient conditions, respectively, in subsequent experiments. Both N conditions were used in our previous report for N limitation analysis in Arabidopsis [33]. The epidermal cell length in *atdfb-3*, as measured using a field emission scanning electronic microscope (FE-SEM), was approximately 86% compared with the wild-type cell length on 9.4 N (396.8±60.6 µm and 462.0±35.8 µm, respectively) and 47% compared with the wild-type cell length on 0.3 N (226.2±36.5 µm and 484.2±35.6 µm, respectively) (Figures 5D and E). These observations demonstrated that *atdfb-3* was defective in hypocotyl cell elongation in darkness.

To explore why *atdfb-3* was sensitive to external NO_3_^−^ concentrations, the stage at which N-sufficient conditions were required for *atdfb-3* hypocotyl development was investigated by removing NO_3_^−^ from the medium at various time points after sowing. Seedlings first grown on 9.4 N for 0 to 6 days were transferred to 0.3 N for the remaining days, for a total growth time of 6 days. A significant difference in hypocotyl length between *atdfb-3* and the wild type was observed when seedlings grown on 9.4 N for 2 days before transferring to 0.3 N (Figure 5F). Hypocotyl length of *atdfb-3* first grown on 9.4 N for 3 days or longer time and then transferred to 0.3 N was similar to that of the mutant grown on 9.4 N for 6 days (Figure 5F). These results indicated that N-sufficient conditions were important for *atdfb-3* during the first 3 days. In further time-course experiments, the hypocotyl length of seedlings grown under N-limited conditions and then transferred to N-sufficient conditions was shorter than those continuously grown on 9.4 N for 6 days and longer than those continuously grown on 0.3 N for 6 days (Figure 5G). The less time *atdfb-3* was grown on 0.3 N before transferring to N-sufficient conditions, the longer the hypocotyls (Figure 5G). The hypocotyl in 6-day-old wild-type seedlings grown on 0.3 N for 2 to 5 days before transferring to 9.4 N was longer than those continuously grown on 0.3 N or 9.4 N for 6 days (Figure 5G); however, this phenomenon was not observed when wild-type seedlings were transferred from N-sufficient to N-limited conditions (Figure 5F). It is possible that the transferring from N-limited to N-sufficient conditions stimulated hypocotyl elongation in the wild type. These results indicated that N-sufficient conditions were required for early growth of *atdfb-3*, and that the response of *atdfb-3* to low N stress occurred within a narrow developmental window (3 days or earlier).

The endosperm in *atdfb-3* was removed under both N conditions to explore whether the storage in embryo or endosperm in *atdfb-3* was altered, which would affect hypocotyl development under dark conditions. When the endosperm was removed, wild-type hypocotyls were slightly shorter than those with endosperm present under both 9.4 N and 0.3 N conditions (Figure 5H). However, the hypocotyls in *atdfb-3* without endosperm under 9.4 N were only 30% of those in *atdfb-3* with the endosperm, and even shorter than those in *atdfb-3* with endosperm under 0.3 N (Figure 5H). Defects in hypocotyl elongation were more significant in *atdfb-3* without endosperm than that with endosperm under both N conditions (Figure 5H). These results indicated that, unlike the wild type, the embryo alone could not satisfy hypocotyl development in *atdfb-3* under N-sufficient conditions, and that the endosperm was vital for *atdfb-3* hypocotyl development. Under N-limited condition, external N could not satisfy hypocotyl development of *atdfb-3* even with the endosperm. The requirement of sufficient NO_3_^−^ during early hypocotyl development in *atdfb-3* could be due to altered seed storage in embryo.

### *AtDFB* was expressed in early developmental stage in Arabidopsis

**Figure 6:**
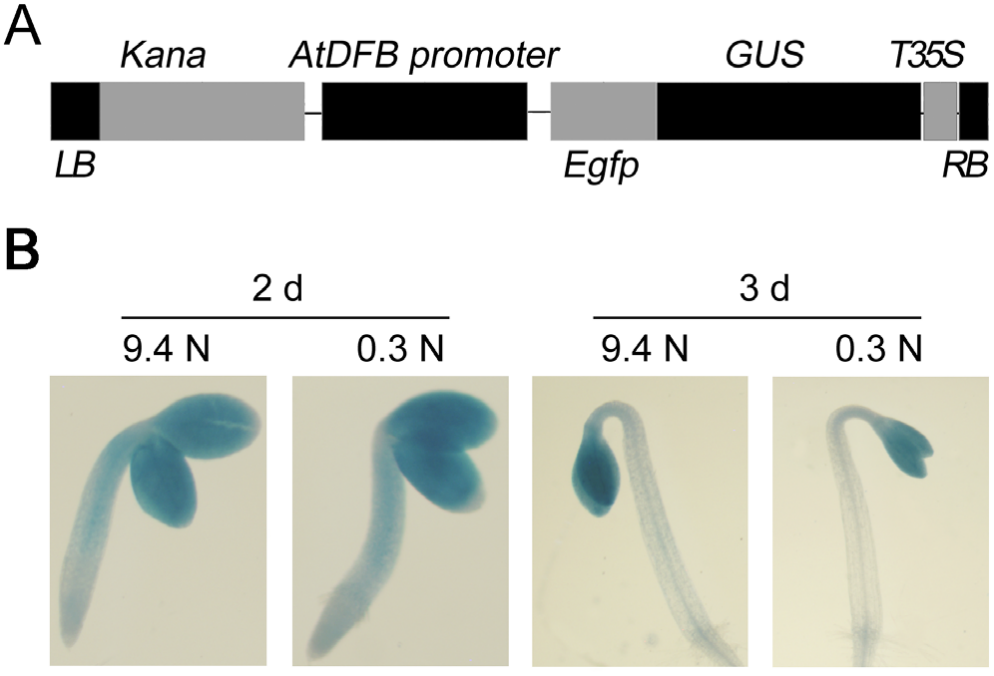
Histochemical localization of *AtDFB* promoter activity and *AtDFB* expression patterns. (A) Schematic diagram of the *AtDFB: GUS* construct. LB and RB indicate the left and right borders, respectively, and Kana indicates the kanamycin resistance gene. (B) GUS staining of 2- and 3-day-old etiolated seedlings under 9.4 N or 0.3 N conditions.

Since *AtDFB* is important for early seedling establishment under dark conditions, we investigated the expression pattern of *AtDFB* during the early stage in etiolated seedlings to illustrate its importance in hypocotyl development. A plasmid containing an *AtDFB* promoter-driven GUS fragment was introduced into the wild-type plants (Figure 6A). Histochemical GUS staining showed that *AtDFB* was strongly expressed in cotyledons and hypocotyls in 2-day-old germinating seeds, while it was strongly expressed only in cotyledons in 3-day-old etiolated seedlings (Figure 6B). The expression pattern of *AtDFB* under light was similar to that in the dark (Figure S3 in File S1). These results indicated that *AtDFB* was expressed in early seedling developmental stage.

### Rescue of *atdfb-3* seedling establishment by exogenous 5-F-THF depended on the nitrate supply

5-F-THF is able to rescue the *atdfb* defects in primary root development in light [27]. We added various concentrations of 5-F-THF or 5-methyl-tetrahydrofolate (5-M-THF) to 0.3 N medium to determine whether folate derivatives could rescue defects in hypocotyl elongation in *atdfb-3* in dark. The difference in hypocotyl length between the mutant and wild-type seedlings was reduced when grown on 0.3 N with 0.5 µM or 5 µM 5-F-THF, while treatment with 50 or 500 µM 5-F-THF rescued hypocotyl elongation in *atdfb-3* seedlings (Figure S4A in File S1). 5-M-THF stimulated hypocotyl elongation in both *atdfb-3* and wild-type seedlings. Interestingly, under N-limited conditions, disparities between the mutant and wild type decreased with increasing 5-M-THF concentrations, but the hypocotyl length in *atdfb-3* was still only 67% of the wild type when grown with 500 µM 5-M-THF (Figure S4B in File S1). These results indicated that 5-M-THF can only partially rescue the hypocotyl elongation in *atdfb-3*. Thus, 50 µM 5-F-THF was chosen to rescue the hypocotyl elongation defect in *atdfb-3* under dark conditions.

**Figure 7:**
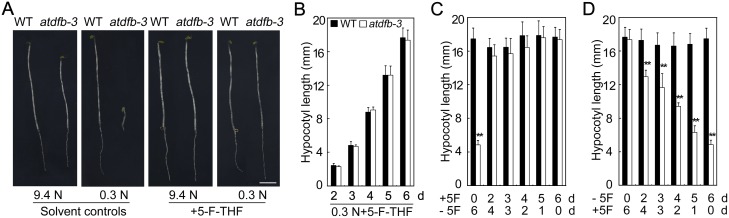
Exogenous 5-F-THF restored the wild-type hypocotyl phenotype in etiolated *atdfb*-3 seedlings. (A) Images and hypocotyl of 6-day-old etiolated WT and *atdfb-3* seedlings germinated on 9.4 N or 0.3 N medium with or without 50 µM 5-F-THF. (B) Hypocotyl length of WT and *atdfb-3* under N-limited conditions with 5-F-THF for various days in darkness. (C) Hypocotyl length of 6-day-old WT and *atdfb-3* etiolated seedlings. Seedlings of both genotypes were grown on 0.3 N medium with 50 µM 5-F-THF for 0 to 6 days and then transferred to 0.3 N medium without 5-F-THF for the remaining time (0 to 6 days). (D) Hypocotyl length of 6-day-old WT and *atdfb-3* etiolated seedlings. Seedlings of both genotypes were grown on 0.3 N medium for 0 to 6 days and then transferred to 0.3 N medium with 50 µM 5-F-THF for the remaining time (0 to 6 days). Data represent means ± SD (n = 15). White bars with ** indicate a highly significant difference at *P*<0.01 (Student’s *t*-test).

The hypocotyl length of 6-day-old *atdfb-3* seedlings was restored to the wild-type level under both N-sufficient and N-limited conditions with 50 µM 5-F-THF (Figure 7A). Further analysis indicated that 5-F-THF could rescue the hypocotyl elongation defects in *atdfb-3* seedlings under N-limited conditions at various experimental time points (Figure 7B). We next examined the stages at which folate was vital for hypocotyl elongation. Both *atdfb-3* and the wild type seeds were grown on N-limited conditions with 5-F-THF for 0 to 6 days and then moved to conditions without 5-F-THF for the remaining time, for a total growth time of 6 days. We found that the mutant seedlings grown on medium with 5-F-THF for only 2 days and then transferred to conditions without 5-F-THF could adapt to low-N conditions, demonstrating the same hypocotyl length as the wild type at day 6 (Figure 7C). However, *atdfb-3* seedlings grown on medium with 5-F-THF for 1 day before transferring to conditions without 5-F-THF could not adapt to N-limited conditions, similar to those without 5-F-THF treatment under N-limited conditions (Figure S4C in File S1). Additionally, the mutant seedlings grown on medium without 5-F-THF for 2 or more days before transferring to medium with 5-F-THF showed shorter hypocotyls than did the wild-type seedlings; the longer time the mutant was grown on medium without 5-F-THF before transferring to medium with 5-F-THF, the shorter the hypocotyls (Figure 7D). These results suggested that intact folate metabolism was necessary for early (2 days or earlier) developmental stages in Arabidopsis.

When no N was applied to the medium, 5-F-THF could not rescue hypocotyl defects in *atdfb-3*. Meanwhile, 5-F-THF could not restore hypocotyl defects in *atdfb-3* when NH_4_^+^ was the sole N source in the medium (Figure S5A and B in File S1). These results indicated that the recovery of hypocotyl development in *atdfb-3* by 5-F-THF depended on exogenous NO_3_^−^ supply.

### Folate metabolism was altered in *atdfb-3* germinating seeds and etiolated seedlings

To increase our understanding of how folate metabolism was perturbed in the mutant, liquid chromatography/mass spectroscopy (LC-MS) was employed to profile various folate derivatives in early developmental stage of 2-day-old germinating seeds. We found that 5-M-THF was the major folate derivative, accounting for 70% of the total folates (Figure S6 and Table S1 in File S1). Under N-sufficient conditions, the mutant contained less 5-F-THF, 5-M-THF, and total folates than the wild type (approximately 30%, 80%, and 75% of the wild type, respectively). Under N-limited conditions, the contents of most folate derivatives decreased in the wild type, e.g. 67% reduction for 5-F-THF and 35% for 5-M-THF, respectively, but remained unchanged in *atdfb-3* (Figure S6 and Table S1 in File S1).

**Figure 8:**
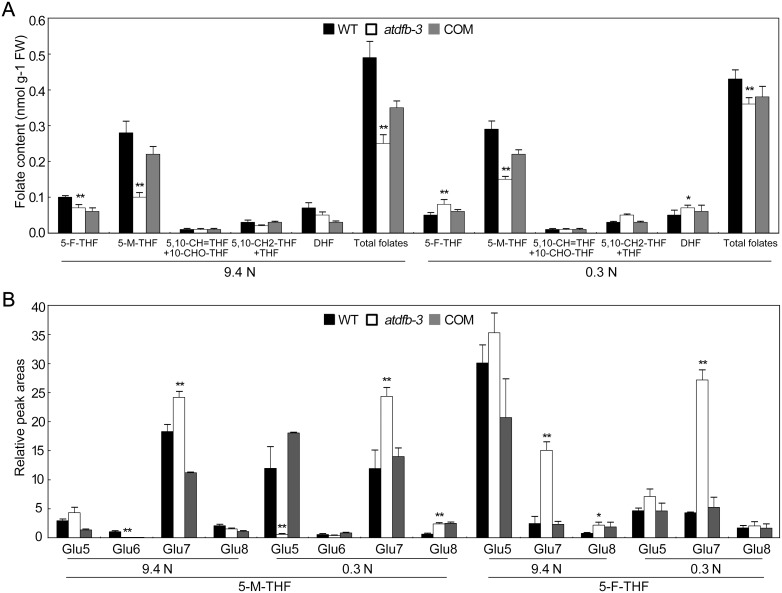
Folate profiles in 6-day-old WT, *atdfb-3*, and *AtDFB* complemented (COM) seedlings grown on 9.4 N or 0.3 N medium. (A) Levels of individual folates and total folates in seedlings. The folate species detected were: 5-formyl-THF (5-F-THF), 5-methyl-THF (5-M-THF), 5,10-methenyl-THF (5,10-CH = THF), 10-formyl-THF (10-CHO-THF), 5,10-methylene THF (5,10-CH_2_-THF), tetrahydrofolate (THF), and dihydrofolate (DHF). Note that 10-formyl-THF (10-CHO-THF) and 5,10-CH = THF are grouped together and THF and 5,10-methylene THF (5,10-CH_2_-THF) are grouped together because the procedure used for folate analysis results in inter-conversion of these pairs of folate species. (B) Relative LC-MS peak areas of folylpolyglutamates (5-M-THF-Glu_n_ and 5-F-THF-Glu_n_, n = 5, 6, 7, or 8). Data are means ± SD (n = 5). Each replicate consisted of 100 mg of pooled plant material. A significant difference at *P*<0.05 is indicated by *, and a highly significant difference at *P*<0.01 is indicated by ** (Student’s *t*-test).

To further determine how the *AtDFB* mutation interferes with folate metabolism in dark-grown seedlings, various folate derivatives and poly-glutamylated 5-M-THF and 5-F-THF were examined in 6-day-old etiolated seedlings (Figure 8). First, we found that there was a difference in folate derivative contents between the mutant and wild type. Under N-sufficient conditions, the mutant contained less 5-F-THF, 5-M-THF, and total folates than the wild type (approximately 70%, 36%, and 51% of the wild type, respectively). 5-M-THF, the major folate derivative, constituted the major deficiency in total folates in *atdfb-3* seedlings. Under N-limited conditions, higher accumulation of folate derivatives including 5-F-THF and DHF was observed in *atdfb-3* than that of the wild type, which was opposite to that under N-sufficient conditions; however, 5-M-THF remained less in *atdfb-3* than in the wild type (Figure 8A and Table S2 in File S1). Second, we found that the folate derivatives profiling of the mutant and wild type responded differentially to N limitation. For example, N limitation led to a 50% decrease in 5-F-THF in the wild type, but had no effects on *atdfb-3*, resulting in a 1.6-fold accumulation of 5-F-THF in *atdfb-3* as compared to the wild type. N limitation led to no significant decrease in total folates in the wild type, but a 44% increase in *atdfb-3* seedlings, resulting in a drastic reduction of the difference from 49% to 16% between the mutant and wild type (Figure 8A and Table S2 in File S1).

The levels of polyglutamated folates with 5-, 6-, 7-, and 8-Glu tails were compared between the mutant and wild type based on relative peak areas due to a lack of standards. There was a significant difference between the two genotypes. Under N-sufficient conditions, most striking difference was observed for both 5-M-THF-Glu7 and 5-F-THF-Glu7, i.e. higher accumulation in *atdfb-3* than the wild type. In addition, 5-M-THF-Glu6 was less and 5-F-THF-Glu8 was higher in *atdfb-3* than that of the wild type, respectively (Figure 8B). Under N-limited conditions, both 5-M-THF-Glu7 and 5-F-THF-Glu7 remained higher in the mutant than in the wild type as observed under N-sufficient conditions. 5-M-THF-Glu5 and 5-M-THF-Glu8 was around 19 folds less and 2.9 folds higher in *atdfb-3* than in the wild type, respectively (Figure 8B). Moreover, the folate derivatives with polyglutamates in the mutant and wild type differed in responding to N limitation. For example, N limitation led to a 3.1-fold increase of 5-M-THF-Glu5 in the wild type, but a 6.1-fold decrease in *atdfb-3*. The pattern of the folylpolyglutamation profile in the complemented transformants was similar to that in the wild type (Figures 8B).

We also analyzed the expression of genes involved in folate biosynthesis and C1 metabolism (Figure S7 in File S1). The expression of *AtDFA* and *AtDFC* were enhanced in 2-day-old *atdfb-3* seedlings due to loss function of *AtDFB* especially under 0.3 N, but not in 6-day-old seedlings (Figure S7A and B in File S1). Most of these genes, including *AMINODEOXYCHORISMATE LYASE* (*ADCL*), *10-FORMYL-THF DEFORMYLASE 2* (*FDF2*), *5-FORMYL-THF CYCLOLIGASE* (*5-FCL*), *10-FORMYL-THF SYNTHETASE* (*THFS*), *γ-GLUTAMYL HYDROLASE 1* (*GGH1*) and *GGH2*, showed higher expression in *atdfb-3* than in the wild type under both N conditions. Low N stimulated the expression of *ADCL*, *PDF2* and *MTHFR2* in both genotypes, but only that of *5-FCL* in the mutant and *THFS* in the wild type, respectively (Figure S7C in File S1).

### N metabolism was affected in *atdfb-3* germinating seeds and etiolated seedlings

**Figure 9:**
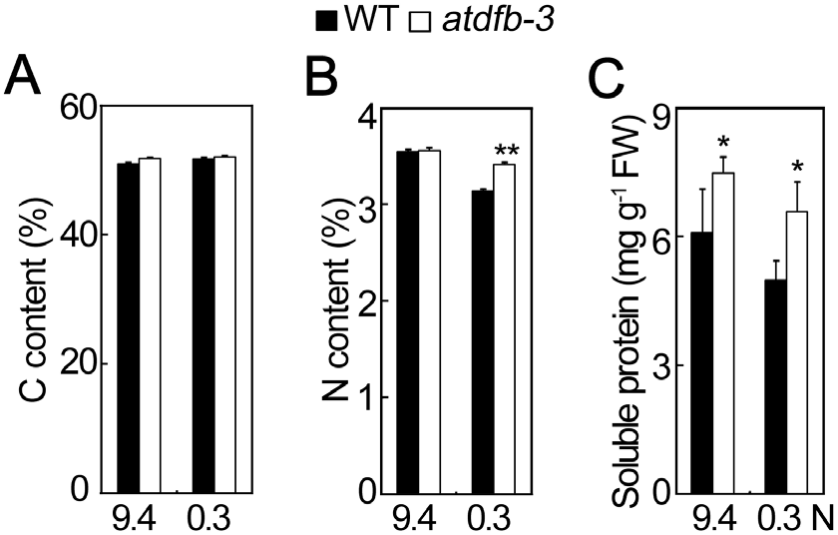
N Metabolites in 2-day-old WT and *atdfb-3* germinating seeds under 9.4 N or 0.3 N. (A) C content. (B) N content. (C) Soluble protein content. Data represent means ± SD. A–B, n = 4, and each replicate consisted of 10 mg DW pooled plant material; C, n = 3, and each replicate consisted of 50 mg pooled plant material. A significant difference at *P*<0.05 is indicated by *, and a highly significant difference at *P*<0.01 is indicated by ** (Student’s *t*-test).

Under N-sufficient conditions, the C and N contents in *atdfb-3* were unchanged compared with the wild type, while the N content in *atdfb-3* increased by 9% under N-limited conditions (Figure 9A and B). In addition, the mutant accumulated 23% more soluble protein under 9.4 N and 32% more under 0.3 N than did the wild type (Figure 9C). These results indicated that N metabolism in germinating *atdfb-3* seeds was altered under N-limited conditions.

**Figure 10:**
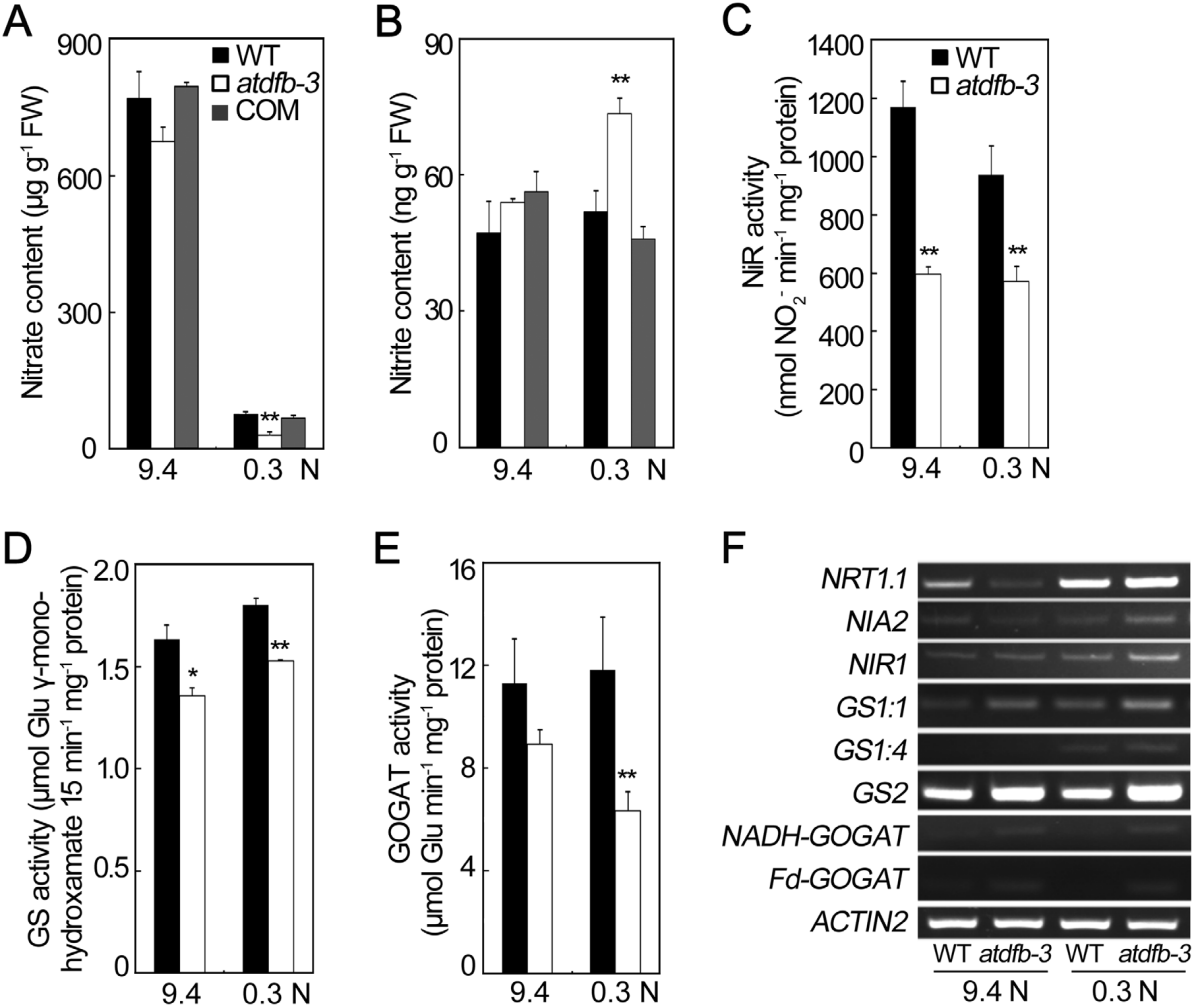
Altered biochemical characteristics of N reduction and assimilation in 6-day-old etiolated WT, *atdfb-3* and *AtDFB* complemented (COM) seedlings on 9.4 N or 0.3 N medium. (A) NO_3_^−^ content. (B) NO_2_^−^ content. (C) NiR activity. (D) GS activity. (E) GOGAT activity. (F) Altered transcript levels of genes involved in nitrate transport, reduction, and N assimilation. Data are means ± SD (n = 3). In panel (A) and (B), each replicate consisted of 1 g of pooled plant material. In panel (C) and (D), each replicate consisted of 200 mg of pooled plant material. In panel (E), each replicate consisted of 500 mg of pooled plant material. A significant difference at *P*<0.05 is indicated by *, and a highly significant difference at *P*<0.01 is indicated by ** (Student’s *t*-test).

Because *atdfb-3* showed a failure of seedling establishment when grown on low N, we sought to understand how N metabolism was affected by the *AtDFB* mutation by analyzing N-relating metabolites and enzyme activities. Under N-sufficient conditions, there was no significant difference in NO_3_^−^ and NO_2_^−^ contents between *atdfb-3* and the wild type; however, under N-limited conditions lower level of NO_3_^−^ and higher level of NO_2_^−^ were detected in *atdfb-3* than in the wild type (Figure 10A and B), whereas no significant difference in NH_4_^+^ contents was observed (Figure S8 in File S1). Subsequently, activities of the enzymes involved in N metabolism, such as nitrite reductase (NiR), glutamine synthetase (GS), and glutamine 2-oxoglutarate amino transferase (GOGAT) were investigated under both N conditions. NiR activity was lower in *atdfb-3* than in the wild type under both 9.4 N and 0.3 N, about 51% and 61% of the wild type, respectively (Figure 10C). Under 9.4 N or 0.3 N, GS activity in *atdfb-3* was 83% or 85% of the wild type, respectively (Figure 10D). The GOGAT activity did not differ significantly between *atdfb-3* and the wild type under 9.4 N, while that in *atdfb-3* was only half of the wild type under 0.3 N (Figure 10E). These results implied that activity of the enzymes involved in N reduction and assimilation was altered in *atdfb-3* etiolated seedlings.

Next, transcripts of genes involved in NO_3_^−^ transport, NO_3_^−^ reduction, and N assimilation were also examined in *atdfb-3*. When N was sufficient, *atdfb-3* had lower level of expression of *NITRATE TRANSPORTER 1.1* (*NRT1.1*) than the wild type; however, transcripts of *GS1∶1* and *GS2* were more abundant in *atdfb-3* than in the wild type. Low-N stress increased the expression level of *NRT1.1* and *GS1∶4* in both genotypes (Figure 10F). Taken together, these results indicated that N metabolism was perturbed in *atdfb-3*.

## Discussion

Under light conditions, mutation in the *AtDFB* resulted in a short primary root due to perturbed folate profile [32]. In this report, *atdfb-3* was characterized for its defects in seed reserves and hypocotyl elongation in the dark due to loss of function of *AtDFB*, providing novel insights into a potential link among folate metabolism, seed reserves, and hypocotyl development in Arabidopsis.

### *AtDFB* mutation altered seed storage

Seed storage compound synthesis and accumulation in maturing seeds of Arabidopsis are under the control of many factors, such as hormones, sugars, master regulator genes, and transcription factors [4]. Folates also play an important role in seed development, since *gla1* and *fpgs1fpgs2* exhibited defective embryo development [30], [34]. The double knockout (dKO) mutation of 10-formyl-THF deformylase resulted in shriveled seeds and low amounts of lipids, such as 20∶1 [31]. A slightly but significantly lower level of 20∶1, one of the markers for storage oil in Arabidopsis [35], was also detected in *atdfb-3* than in the wild type (Figure 3), probably indicative of a low oil storage in the mutant.

Arabidopsis mutants that have defective seed storage mobilization had shorter hypocotyls in the dark [16], [17], and *atdfb-3* also showed shortened hypocotyls (Figure 5A). There is a possibility that high level of mannose in *atdfb-3* seeds reduced storage mobilization rate, as exogenous mannose did greatly reduce the rate of storage lipid mobilization in germinating Arabidopsis seeds [36]. It was also reported that galactose that accumulated during seed maturation could provide easily available energy and also be an important component of the sugar signaling pathway during germination of pea seeds [9], [37]. Thus, high content of galactose accumulated in *atdfb-3* seeds might promote germination while mannose inhibits storage mobilization. Besides, mannose and galactose are intermediates of ascorbic acid biosynthesis [38]. Therefore, it needs further investigation that whether the accumuation of mannose and galactose is due to deficient ascorbic acid biosynthesis in *atdfb-3*.

It was also observed that oxalic acid was accumulated in *atdfb-3* seeds (Figure 3). Oxalate could be produced by glycolate or glyoxylate during photorespiration, or by the breakdown of ascorbic acid [39], [40]. Previously we reported that the mitochondrial AtDFC is involved in regulation of N metabolism in Arabidopsis by linking folate metabolism with photorespiration [33]. However, it seems unlikely that the oxalate accumulation is due to photorespiration alteration given the fact that Gly/Ser ratio, an indicator for photorespiration, was not changed in the mutant (Figure 4C). Oxalate accumulation was observed both in *atdfb-3* and the mutant of oxalyl-CoA synthetase, an enzyme that catalyzes the first step of oxalate catabolism [41]. However, it remains unclear that whether the oxalate accumulation in *atdfb-3* seeds is attributable to decreased oxalate catabolism. Pentanedioic acid was dramatically increased in the *atdfb-3* seeds (Figure 3), but it is unknown yet how folates affect its biosynthesis in plants to date. In addition, altered levels of many individual amino acids in *atdfb-3* verified the role of folates in amino acids metabolism [31], [32], [33], [42], [43].

### Folate biosynthesis and polyglutamylation were responsive to low N stress

Low N stress enhanced the expression of folate synthesis and metabolism genes (such as *AtDFA* and *THFS*) in the mutant (Figure S7A in File S1). As a result, similar contents of total folates in the mutant and wild type were achieved (Figure S6 in File S1). This is different from our previous report that the total folates level in *atdfc* remained lower than in the wild type under N limitation [33]. Under N-sufficient conditions, hypocotyls of 6-day-old etiolated *atdfb-3* seedlings were only slightly shorter than the wild type; however, under N-limited conditions, the mutant had significantly reduced hypocotyls (Figure 5A and B). In association with this, 5-M-THF-Glu5 and 5-F-THF-Glu7 in *atdfb-3* was 0.05- and 6-fold of that in the wild type under N-limited conditions, respectively (Figure 8B). Considering that 5-M-THF-Glu5 and 5-F-THF-Glu7 were the most changed folate derivative and responded in an opposite manner when the mutant was subjected to low N, we hypothesize that 5-M-THF-Glu5 and 5-F-THF-Glu7 may, at least in part, play an important but contrasting role in regulation of hypocotyl development. Meanwhile, we could also speculate that there is not a causal relationship between total folate level and shortened hypocotyl length in the mutant under N limitation because the mutant and wild type had a similar level of total folates (Figure 8A and S6).

### Exogenous nitrate was required for the rescue of *atdfb-3* mutant by 5-F-THF application

Seed reserves are mobilized to fuel seedlings until autotrophic growth. Given the massive reduction in nitrate level in *atdfb-3* (Figure 4D), it was reasonable that the mutant requires sufficient external N. 5-F-THF could rescue the defects in hypocotyl elongation in *atdfb-3* only in the presence of NO_3_^−^, but not NH_4_^+^ in the medium (Figure S5 in File S1). Owing to the observation of reduction of GS and GOGAT activity under N-limited conditions (Figure 10), NH_4_^+^ assimilation is probably deficient in *atdfb-3*. Thus, the mutant could not utilize the sole N source NH_4_^+^ to fuel seedling development. Additionally, exogenous 5-F-THF failed to rescue the mutant when NO_3_^−^ was absent (Figure S5 in File S1), demonstrating an absolute necessity of NO_3_^−^ for seedling establishment in *atdfb-3*.

The exogenously supplied 5-F-THF was probably absorbed and converted into other active forms of folate by 5-FCL, MTHFR, or other enzymes that convert folate derivatives [44], [45]. The recovery of hypocotyl elongation in *atdfb-3* treated with 5-F-THF was probably due to the excess of folates in the monoglutamylated form, which might be able to complement the mutation by accomplishing the same functions as a small amount of polyglutamylated folates. Given the fact that microtubule cytoskeleton plays a crucial role during hypocotyl elongation [46], and abnormal actin cytoskeleton was observed in *atdfb* primary root [32], it’s conceivable that 5-F-THF may promote hypocotyl cell elongation in *atdfb-3* through regulating cytoskeleton stabilization. Taken together, these observations suggest that folate-regulated N metabolism is important for *atdfb-3* seedling development in the dark.

### Perturbation of N metabolism was caused by *atdfb* mutation

Defective hypocotyl elongation in *atdfb-3* is accompanied by perturbed N metabolism due to loss function of *AtDFB*. Under N-sufficient conditions, activities of NiR and GS were both lower in *atdfb-3* than those of the wild type, indicative of an impaired N reduction and assimilation, although the contents of NO_3_^−^, NO_2_^−^ and NH_4_^+^ in *atdfb-3* were similar to those in the wild type (Figure 10 and Figure S8 in File S1). Under N-limited conditions, lower content of NO_3_^−^ may reflect a decreased NO_3_^−^ uptake and/or reduction in *atdfb-3* (Figure 10A), and the lower NiR activity could partly explain why high content of NO_2_^−^ accumulated in *atdfb-3* (Figure 10B and C). Apart from this, low activities of GS and GOGAT were also observed under 0.3 N (Figure 10D and E), suggesting a reduced N assimilation ability in *atdfb-3* as compared to the wild type. Taken together, the ability of N reduction and assimilation in *atdfb-3* was significantly lower than in the wild type under N limitation, resulting in defective hypocotyl development. Furthermore, it was reported that folates can be oxidized by NO_2_^−^ to several pterin products [47], therefore it is possible that insufficient folate derivatives could not effectively protect the etiolated seedling from toxicity of significantly accumulated NO_2_^−^ under N-limited conditions. Thus, the drastically shortened hypocotyl of *atdfb-3* under 0.3 N could be partly due to the NO_2_^−^ toxicity. However, how the altered folates profiling or polyglutamylation affects N metabolism in *atdfb-3* awaits further investigation. One of the possibilities is that polyglutamylated 5-M-THF or 5-F-THF may act as a regulator of the N metabolism enzymes.

Taking together, we provide genetic evidence that the plastidial isoform FPGS is required for normal seed reserve accumulation and hypocotyl elongation under dark conditions. The *AtDFB* mutation results in altered seed storage, perturbed folate profile, altered N metabolism and shorter hypocotyls in etiolated seedlings, and exogenous 5-F-THF recovered shortened hypocotyls of the mutant to the wild-type level when NO_3_^−^ was present in the growth conditions. However, the underlying mechanism through which folate regulates seed reserve accumulation and hypocotyl development during skotomorphogenesis as well as the relationship among folate metabolism, N metabolism and hypocotyl development require further investigation.

## Materials and Methods

### Plant materials and growth conditions

Arabidopsis wild-type (*Arabidopsis thaliana*, ecotype Columbia), the T-DNA insertion mutant of *AtDFB* (SALK_015472, called *atdfb-3* in this report), and the *AtDFB* complemented line were grown in identical growth chambers under a 16-h photoperiod (photosynthetic photon flux density 60 µE m^−2 ^s^−1^) and a day/night temperature of 22/16°C before being harvested. For biochemical analysis, and metabolite measurement assays, seeds from various genetic backgrounds were harvested at the same time and were after-ripened for 3 months.

For the Petri dish-based N limitation experiments, when NO_3_^−^ was used as the sole N source, NH_4_^+^ was removed from the half-strength MS medium [48]. The K^+^ level was balanced with KCl to maintain 9.4 mM K^+^. In this report, 9.4 mM NO_3_^−^ (9.4 N) was used as the N-sufficient condition and 0.3 mM NO_3_^−^ (0.3 N) as the N-limited condition. When NH_4_^+^ was used as the sole N source, NO_3_^−^ was removed from the half-strength MS medium, and NH_4_Cl was then added to the desired N concentration. When Asn and Gln were used as the sole N sources, NO_3_^−^ and NH_4_^+^ were removed from the half-strength MS medium, and the K^+^ level was balanced with KCl to maintain 9.4 mM K^+^. For C- or P-deficiency experiments, sucrose or KH_2_PO_4_ was not added to half-strength MS medium. Endosperm/seed coat tissues were removed from the embryo using a dissecting microscope, after allowing seeds to soften in water for 6 h at 4°C [17]. For all experiments mentioned above, the wild-type and *atdfb-3* seeds were sterilized, grown on the same plate, treated at 4°C in the dark for 2 days, and then moved to a growth chamber at 22°C under continuous dark conditions. Digital photographs of hypocotyls at various stages of etiolated seedling growth were acquired using a Nikon 700 camera, and their lengths were measured using ImageJ.

For expression pattern analysis, 1,406 bp of the *AtDFB* promoter was amplified and cloned into the binary vector pKGWFS 7.0. The construct was introduced into wild-type plants using the floral dipping method. The homozygous *AtDFB: GUS*-transformed seedling were stained according to Francisco [49], and observed under the stereoscope from Nikon DIGITAL CAMERA Dxm 1200F.

For the 5-F-THF and 5-M-THF [(6R, S)-5-formyl-5,6,7,8-tetrahydrofolic acid and (6R, S)-5-methyl-5,6,7,8-tetrahydrofolic acid, calcium salt; Schircks Laboratories, Switzerland] supplementation experiments, a stock solution was added to the growth medium to achieve the desired working concentration. A stock solution of 5 mM 5-F-THF or 5-M-THF was prepared in deionized water. Seeds were planted directly on the medium with or without the abovementioned folate derivatives. The hypocotyl length assays were performed as described above.

### Microscopic analysis

The hypocotyls of 6-day-old etiolated wild-type and *atdfb-3* seedlings grown on 9.4 N or 0.3 N were observed according to the method by Cowling *et al*. under a Hitachi S1-4800 high-resolution FE-SEM [50]. The cells of the midportions of hypocotyls were observed using a ZEISS Imager M1 DIC microscope and a 10× objective lens [51].

### Biochemical analysis

Biochemical analysis procedure was according to Jiang *et al*. [33]. C and N contents were analyzed using a Perkin Elmer 2400 Series II CHNS/O Elemental Analyzer (www. perkinelmer.com), and the value indicated the percentage of C or N in total dry weight (mg/100 mg DW). Free amino acids were analyzed using an ASI.KAUNER amino acid analyzer A200 (www.knauer.net). Soluble proteins were extracted from the frozen seedling powder using 100 mM HEPES-KOH (pH 7.5) and 0.1% Triton X-100 and assayed using a commercial protein assay kit (Bio-Rad). NO_3_^−^ and NO_2_^−^ were measured as described by Oliveira [39]. NH_4_^+^ was measured according to Andrew *et al*. [52].

### Seed metabolite profile analysis using GC-TOF-MS

Seed metabolite analysis using GC-TOF-MS was performed using a method modified from that described previously [9], [53]. Seeds (approximately 20 mg) were homogenized using a pre-cooled mortar and pestle with liquid nitrogen and extracted in 1.5 ml of a methanol: chloroform: water extraction solution (2.5∶1∶1, v/v/v). Internal standards (50 µl 1 mg ml^−1^ ribitol in water and 20 µl 2 mg ml^−1^ C^13^-nonadecanoic acid in chloroform) were subsequently added. The mixture was extracted for 2 h at 37°C with shaking at 1,500 rpm. After 10 min of centrifugation at 12,000 rpm, 400 µl water and chloroform were added to the supernatant, respectively. Following vortexing and a 5-min centrifugation at 12,000 rpm, 200 µl methanol-water phase was isolated and reduced to dryness in a vacuum. Meanwhile, 400 µl chloroform-lipid phase was obtained and concentrated to dryness using nitrogen gas. Residues were re-dissolved and derivatized for 2 h at 37°C (in 25 µl 20 mg ml^−1^ methoxyamine hydrochloride in pyridine) followed by a 30-min treatment with 50 µl N-methyl-N-(trimethylsilyl) trifluoroacetamide at 37°C. Each 1 µl aliquot of the derivatives was injected in a splitless mode using an autosampler into an Agilent 6890 GC system coupled to a LECO Pegasus IV time-of-flight mass spectrometer system (LECO Corporation, USA). A DB-5MS capillary column (30 m×0.25 mm i.d., 0.25-µm film thickness, Agilent J&W Scientific, USA) was used to separate the samples. The injector temperature was 280°C. The Helium gas flow rate through the column was 1.0 ml min^−1^. The column temperature was held at 80°C for 1 min and then increased by 10°C min^−1^ to 310 °C and held there for 10 min. The column effluent was introduced into the ion source of a Pegasus IV TOF-MS. The transfer line and the ion source temperatures were 280 and 200°C, respectively. The electron energy was 70 eV, and mass data were collected in a full-scan mode (m/z 50–650). The detector voltage was set at 1,650 V. All samples were randomized, and five biological replicates were analyzed within 24 h of chemical derivatization. Raw data were processed using LECO ChromaTOF v3.32. Information, including the peak area and retention time, for each detected metabolite was obtained. According to the retention time, the name of each metabolite was obtained by searching the NIST MS Search 2.0 database. When the matching score was higher than 800, the result was considered credible, and the metabolite was further analyzed. The relative contents of the metabolites are shown, and those of the wild type were normalized to values of 1.

### Folate profile analysis using LC-MS

The following folates were purchased from Schircks Laboratories: 5-M-THF, THF, 5-F-THF, 5,10-methenyltetrahydrofolate, and DHF. The 2-day-old etiolated seedlings grown on 9.4 N and 0.3 N medium plates were used for identification of folate profiles. The 6-day-old etiolated seedlings grown on 9.4 N and 0.3 N medium plates were used for identification of folate profiles and folylpolyglutamates of 5-M-THF-Glu_n_ and 5-F-THF-Glu_n_ (n = 5, 6, 7, and 8). The methods for sample preparation and metabolite measurement were described previously [33]. The experiments included five biological replicates.

### NiR, GS and GOGAT enzyme activity analysis

The method for enzyme activity analysis was similar to our previous report [33]. The 200 mg powdered tissues of 6-day-old etiolated seedlings for NiR analysis were added to 0.6 ml of extraction buffer containing 50 mM potassium phosphate buffer (pH 7.5), 1 mM EDTA, 10 mM 2-mercaptoethanol, 100 mM phenylmethanesulfonyl fluoride, and 5 mg PVP and then homogenized. The homogenate was centrifuged, and the supernatant (crude enzyme solution) was used for the NiR activity analysis. A blank sample, in which sulfanilamide was added prior to the extract, was used for background reading. NiR activity was assayed following Takahashi *et al*. [54], with modifications, to measure the decrease of NO_2_^−^ in the assay mixture. A 45 µl sample of the crude enzyme solution was transferred to a 1.5 ml centrifuge tube, and 195 µl of the assay solution containing 50 mM potassium phosphate buffer (pH 7.5), 1 mM NaNO_2_, and 1 mM methyl viologen was added. The reaction was started by adding 60 µl of 57.4 mM Na_2_S_2_O_4_ in 290 mM NaHCO_3_ (final Na_2_S_2_O_4_ concentration in the assay solution, 11.5 mM), and the reaction was run for 5 min at 30°C. A 0.3 ml aliquot was transferred to a new tube containing 0.7 ml water and mixed vigorously to stop the reaction, after which 1 ml 1% (w/v) sulfanilamide in 3 N HCl and 1 ml 0.02% (w/v) *N*-1-naphthylethylenediameine dihydrochloride were added. The absorbance of this mixture at 520 nm was measured. NiR enzyme activity was expressed as nmole NO_2_^−^ used per min per mg protein.

For assessment of total GS activities, freshly harvested samples (500 mg) were ground on ice with 1 ml extraction buffer consisting of 100 mM Tris-HCl (pH 7.6), 1 mM MgCl_2,_ 1 mM EDTA, and 10 mM 2-mercaptoethanol. Semi-synthetase GS activity was assayed, with NH_2_OH used as an artificial substrate, by quantifying the formation of glutamic acid γ-monohydroxamate. The homogenates were centrifuged at 12,000 g for 30 min at 4°C, and the supernatant was analyzed for total GS activities. Total GS activity was measured in a preincubation assay buffer (30°C) consisting of 37.5 mM imidazole buffer (pH 7.0), 30 mM sodium glutamate, 25 mM MgSO_4_, 50 mM NH_2_OH, and 3 mM ATP. The reaction was terminated after 15 min at 30°C by addition of acidic FeCl_3_ solution (88 mM FeCl_3_, 670 mM HCl, and 200 mM trichloroacetic acid). After allowing 10 min for the color development, the reaction mixture was centrifuged at 4,000 g at room temperature for 10 min, and 2 ml of supernatant was then transferred from each well into a new tube. The A_540_ was measured in a spectrophotometer quantification reader [55]. GS enzyme activity was expressed as µmol Glu γ-monohydroxamate formed per 15 min per mg protein.

For assessment of GOGAT activities, freshly harvested samples (200 mg) were ground on ice with 0.6 ml extraction buffer consisting of 100 mM potassium phosphate buffer (pH 7.4), 1.28 mM EDTA, and 10 mM 2-mercaptoethanol. GOGAT activity was assayed by quantifying the formation of Glu and using NADH used as the substrate. The reaction mixture consisted of 100 mM potassium phosphate buffer (pH 7.4), 10 mM Gln, 10 mM 2-oxoglutarate, 0.05 mM NADH, and extract. After a 5-min pre-incubation at 30°C, the reaction was started by adding the reductant solution (1.68 mg Na_2_S_2_O_4_ and 3.48 mg NaHCO_3_ in 1 ml of reaction solution). After a 15 min of incubation at 30°C, the reaction was terminated by heating to 98°C for 5 min. The Glu concentration was then determined using the ninhydrin reaction [56]. GOGAT activity was expressed as µmol Glu formed per min per mg protein.

### Accession numbers

Sequence data from this article can be found at the Arabidopsis Genome Initiative or GenBank/EMBL databases under the following accession numbers: AT5G05980 (*AtDFB*), AT3G18780 (*ACTIN2*), AT1G12110 (*NRT1.1*), AT1G37130 (*NIA2*), AT2G15620 (*NIR1*), AT5G37600 (*GS1∶1*), AT5G16570 (*GS1∶4*), AT5G35630 (*GS2*), AT5G53460 (*NADH-GOGAT*), AT5G04140 (*Fd-GOGAT*), AT5G41480 (*AtDFA*), AT3G10160 (*AtDFC*), AT3G55630 (*AtDFD*), AT5G57850 (*ADCL*), AT5G47435 (*FDF2*), AT5G13050 (*5-FCL*), AT1G50480 (*THFS*), AT1G78660 (*GGH1*), AT1G78680 (*GGH2*), AT2G44160 (*MTHFR2*), and AT3G07270 (*GTPCHI*).

## Supporting Information

File S1 **Contains the following files: Figure S1. Identification of** ***atdfb-3*** **and** ***AtDFB*** **complemented (COM) line.** (A) Gene map of *AtDFB* (At5g05980). Boxes indicate exons and lines indicate introns. T-DNA insertion site for the mutant is indicated. Arrows indicate the positions of the primers (F and R) used for RT-PCR. (B) Schematic diagram of the *ProAtDFB: AtDFB-HWG* complemented construct. LB and RB indicate the left and right borders, respectively, and *Hyg* indicates the hygromycin resistance gene. (C) *AtDFB* transcripts in wild-type (WT), *atdfb*-3, and one representative COM plant. Total RNA was prepared from 14-day-old seedlings grown in light. *ACTIN2* transcripts were used as a loading control. **Figure S2. Hypocotyl phenotypes of 6-day-old etiolated WT and** ***atdfb-3*** **at various concentrations of NH_4_^+^ (A, B) or organic nitrogen (C). Figure S3. GUS staining of 2- and 3-day-old light-grown** ***AtDFB: GUS*** **seedlings under 9.4 N or 0.3 N conditions. Figure S4. Hypocotyl length of WT and** ***atdfb-3*** **under N-limited conditions with 5-F-THF or 5-M-THF treatment.** (A) Hypocotyl length of 7-day-old etiolated WT and *atdfb-3* seedlings after application of various concentrations of 5-F-THF under N-limited conditions. (B) Hypocotyl length of 6-day-old etiolated WT and *atdfb-3* seedlings after application of various concentrations of 5-M-THF under N-limited conditions. (C) Hypocotyl length of 6-day-old WT and *atdfb-3* etiolated seedlings grown on 0.3 N medium with 50 µM 5-F-THF and then transferred to 0.3 N medium without 5-F-THF for the remaining days. **Figure S5. Hypocotyl phenotype of 6-day-old etiolated WT and** ***atdfb-3*** **under 0 N or NH_4_^+^ with 5-F-THF treatment.** (A) Image of hypocotyl phenotype of 6-day-old WT and *atdfb-3* under 0 N or 3 mM NH_4_^+^ with 5-F-THF. (B) Hypocotyl length of 6-day-old WT and *atdfb-3* etiolated seedlings grown on 0 N (upper panel) or 3 mM NH_4_^+^ with 50 µM 5-F-THF (lower panel). **Figure S6. Folate profiles in 2-day-old WT and** ***atdfb-3*** **germinating seeds under 9.4 N or 0.3 N. Figure S7. Transcript levels of genes involved in folate biosynthesis and metabolism. Figure S8. Ammonium content in 6-day-old WT and** ***atdfb-3*** **seedlings in the dark. Table S1.** **Profiles of total folates and various folate species in 2-day-old WT and** ***atdfb-3*** **germinating seeds grown on 9.4 N or 0.3 N medium in the dark. Table S2.** **Profiles of total folates and various folate species in 6-day-old WT,** ***atdfb-3*** **, and** ***AtDFB*** **complemented (COM) etiolated seedlings grown on 9.4 N or 0.3 N medium.**
(DOC)
