# Supplementary material for: Arabidopsis Plastidial Folylpolyglutamate Synthetase Is Required for Seed Reserve Accumulation and Seedling Establishment in Darkness
Source: PLoS One. 2014 Jul 7;9(7):e101905. doi: 10.1371/journal.pone.0101905 (PMC4084893; doi:10.1371/journal.pone.0101905)
Supplement: File S1 — Contains the following files: Figure S1. Identification of atdfb-3 and AtDFB complemented (COM) line. (A) Gene map of AtDFB (At5g05980). Boxes indicate exons and lines indicate introns. T-DNA insertion site for the mutant is indicated. Arrows indicate the positions of the primers (F and R) used for RT-PCR. (B) Schematic diagram of the ProAtDFB: AtDFB-HWG complemented construct. LB and RB indicate the left and right borders, respectively, and Hyg indicates the hygromycin resistance gene. (C) AtDFB transcripts in wild-type (WT), atdfb-3, and one representative COM plant. Total RNA was prepared from 14-day-old seedlings grown in light. ACTIN2 transcripts were used as a loading control. Figure S2. Hypocotyl phenotypes of 6-day-old etiolated WT and atdfb-3 at various concentrations of NH4+ (A, B) or organic nitrogen (C). Figure S3. GUS staining of 2- and 3-day-old light-grown AtDFB: GUS seedlings under 9.4 N or 0.3 N conditions. Figure S4. Hypocotyl length of WT and atdfb-3 under N-limited conditions with 5-F-THF or 5-M-THF treatment. (A) Hypocotyl length of 7-day-old etiolated WT and atdfb-3 seedlings after application of various concentrations of 5-F-THF under N-limited conditions. (B) Hypocotyl length of 6-day-old etiolated WT and atdfb-3 seedlings after application of various concentrations of 5-M-THF under N-limited conditions. (C) Hypocotyl length of 6-day-old WT and atdfb-3 etiolated seedlings grown on 0.3 N medium with 50 µM 5-F-THF and then transferred to 0.3 N medium without 5-F-THF for the remaining days. Figure S5. Hypocotyl phenotype of 6-day-old etiolated WT and atdfb-3 under 0 N or NH4+ with 5-F-THF treatment. (A) Image of hypocotyl phenotype of 6-day-old WT and atdfb-3 under 0 N or 3 mM NH4 + with 5-F-THF. (B) Hypocotyl length of 6-day-old WT and atdfb-3 etiolated seedlings grown on 0 N (upper panel) or 3 mM NH4 + with 50 µM 5-F-THF (lower panel). Figure S6. Folate profiles in 2-day-old WT and atdfb-3 germinating seeds under 9.4 N or 0.3 N. Figure S7. Tra [file pone.0101905.s001.doc]

**Supplemental Information**

## RT-PCR and Real-Time RT-PCR expression analysis

Total RNA was isolated from whole seedlings using TRIzol reagent (Invitrogen). To eliminate any residual genomic DNA, total RNA was treated with RNase-free DNase I (NEB), and the RevertAid First Strand cDNA Synthesis kit (Fermentas) was used to synthesize first-strand complementary DNAs (cDNAs). RNA transcripts were subjected to RT-PCR and examined on ethidium bromide-stained agarose gels. *ACTIN2* was used as a control.

For relative quantiﬁcation values for each target gene were calculated by the 2^-ΔΔCT^ method. *ACTIN2* was used as a internal control, and control treatment (9.4 N WT seedlings) was normalized to a value of 1. Primers are as followed:

*ACTIN2*-F: ATGTCTCTTACAATTTCCCG;

*ACTIN2*-R: CCAACAGAGAGAAGATGACT;

*AtDFB*-F: CGTTGGCTGCTTTGTCGTCT;

*AtDFB*-R: ATTCCAGCCTTCTCACCAGCA;

*AtDFA*-F: TCACTGGGATAGCCTTCTC;

*AtDFA*-R: CACTGGGCGACCGTGTTTA;

*AtDFC*-F: GCAATTCAGACTACAAAGACTATGG;

*AtDFC*-R: CTGTGACCAGAACCTCTA;

*AtDFD*-F: GAGTGGAGTTCCTGCTTTTACAGTGGC;

*AtDFD*-R: TGTAGAGCACAACGCAACAGCAAGA;

*ADCL-F*: GGTAGTGACCTCGTCCATCC;

*ADCL-R*: TAACAACAAACGCCACATT;

*FDF2-F*: TCAGGATGCTGTTGGGATT;

*FDF2-R*: TCATCCACTTGGCTTCTCG;

*5-FCL-F*: GCGTTTGACAGATGTGGGC;

*5-FCL-R*: AGGTGTCACAAGGGCATCG;

*THFS-F*: GCGAGAATGTTTCCTTAGTTG;

*THFS-R*: CCAATGTCCAATGGGTAG;

*GGH1-F*: GAACTTTACTCGTCGTCTTGTGTGTT;

*GGH1-R*: ACAAGGGAAATCATGCAAGACA;

*GGH2-F*: TTCGACTCTGTTATTATCTGTGAACTTG;

*GGH2-R*: GATAGAAAAGCGTACATTATCTAGTTTAATGTC;

*MTHFR2-F*: TTGCGTATGACTGGTTTCTG;

*MTHFR2-R*: GGGCGTCTCCAGGGTAAAG;

*GTPCHI-F*: TGTCAATCGGGAAGGCATC;

*GTPCHI-R*: CCCGATGGGACATAACCT;

*NRT1.1-F*: AGGAACGGGAGGCGTGAAG;

*NRT1.1-R*: ACCTGCGTCATCGGGCTAC;

*NIA2-F*: AGGAGATTCTTCCCATCAACG;

*NIA2-R*: CCAACACCAGAACTTCCCATAC;

*NIR1-F*: AGGAAGTATGGTGAAGATGGGTG;

*NIR1-R*: GCGGTGATAAACTGCGAAAG;

*GS1:1-F*: TCTCGGCTGCTGATGAAAT;

*GS1:1-R*:CCTTCTTCCCTCATTGACTTG;

*GS1:4-F*: GAGTTGGAGCAGACAAAGCC;

*GS1:4-R*: TGTAACGAGCAACCCAGACC;

*GS2-F*: AACCGTGGATGCTCTATTCG;

*GS2-R*: AAGAGTTGGCTCCCACAGG;

*NADH-GOGAT-F*: CTGGGATTCTTGTTGGTCTG;

*NADH-GOGAT-R*: CGGTAGGCACTAATCTCC;

*Fd-GOGAT-F*: TTAATTGAAGCACATGTGGAAAA;

*Fd-GOGAT-R*: TTGCCAGAAGAGAGGTAGATACTTT.


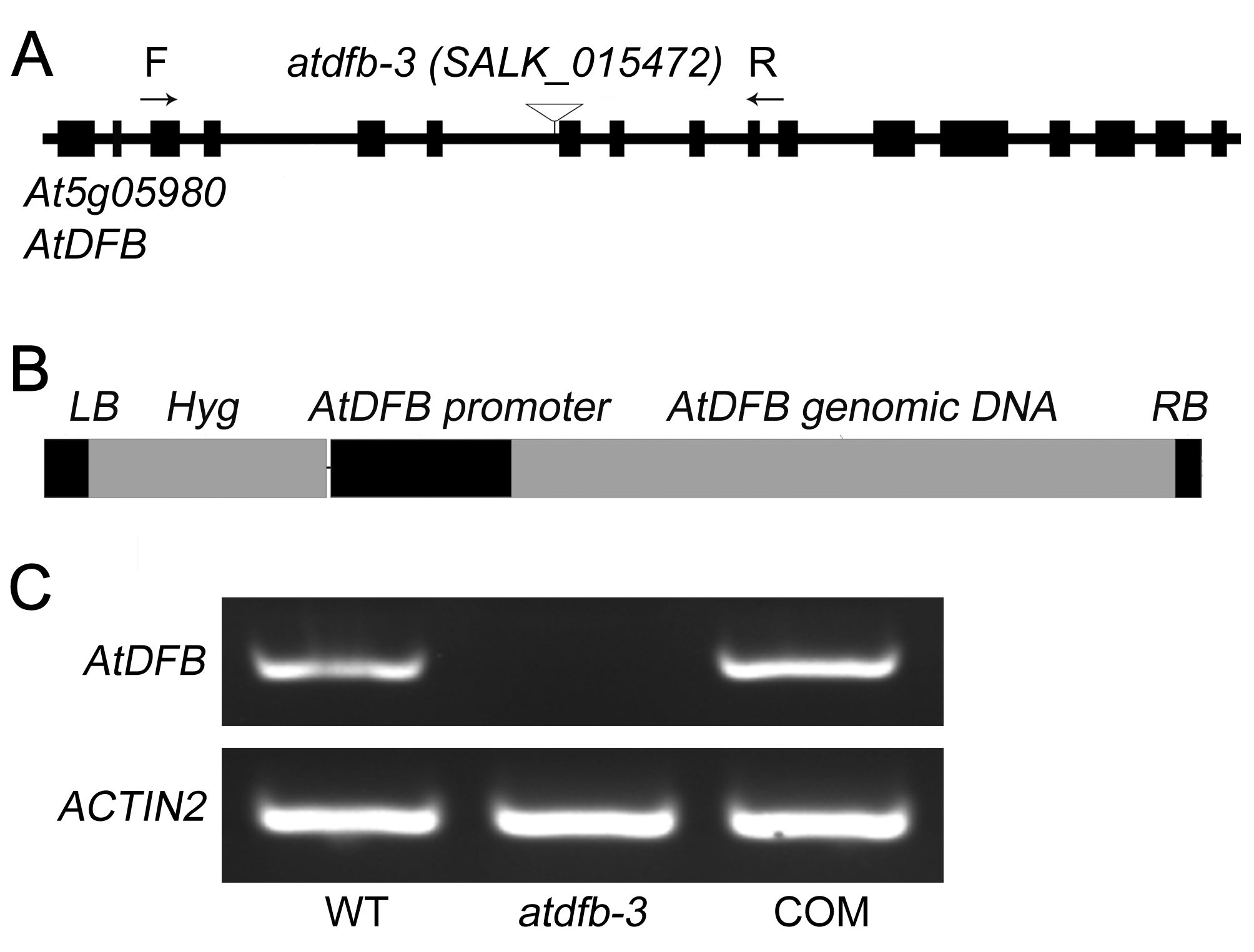


**Figure S1.** Identification of *atdfb-3* and *AtDFB* complemented (COM) line. (A) Gene map of *AtDFB* (At5g05980). Boxes indicate exons and lines indicate introns. T-DNA insertion site for the mutant is indicated. Arrows indicate the positions of the primers (F and R) used for RT-PCR. (B) Schematic diagram of the *ProAtDFB:AtDFB-HWG* complemented construct. LB and RB indicate the left and right borders, respectively, and *Hyg* indicates the hygromycin resistance gene. (C) *AtDFB* transcripts in wild-type (WT), *atdfb*-3, and one representative COM plant. Total RNA was prepared from 14-day-old seedlings grown in light. *ACTIN2* transcripts were used as a loading control.


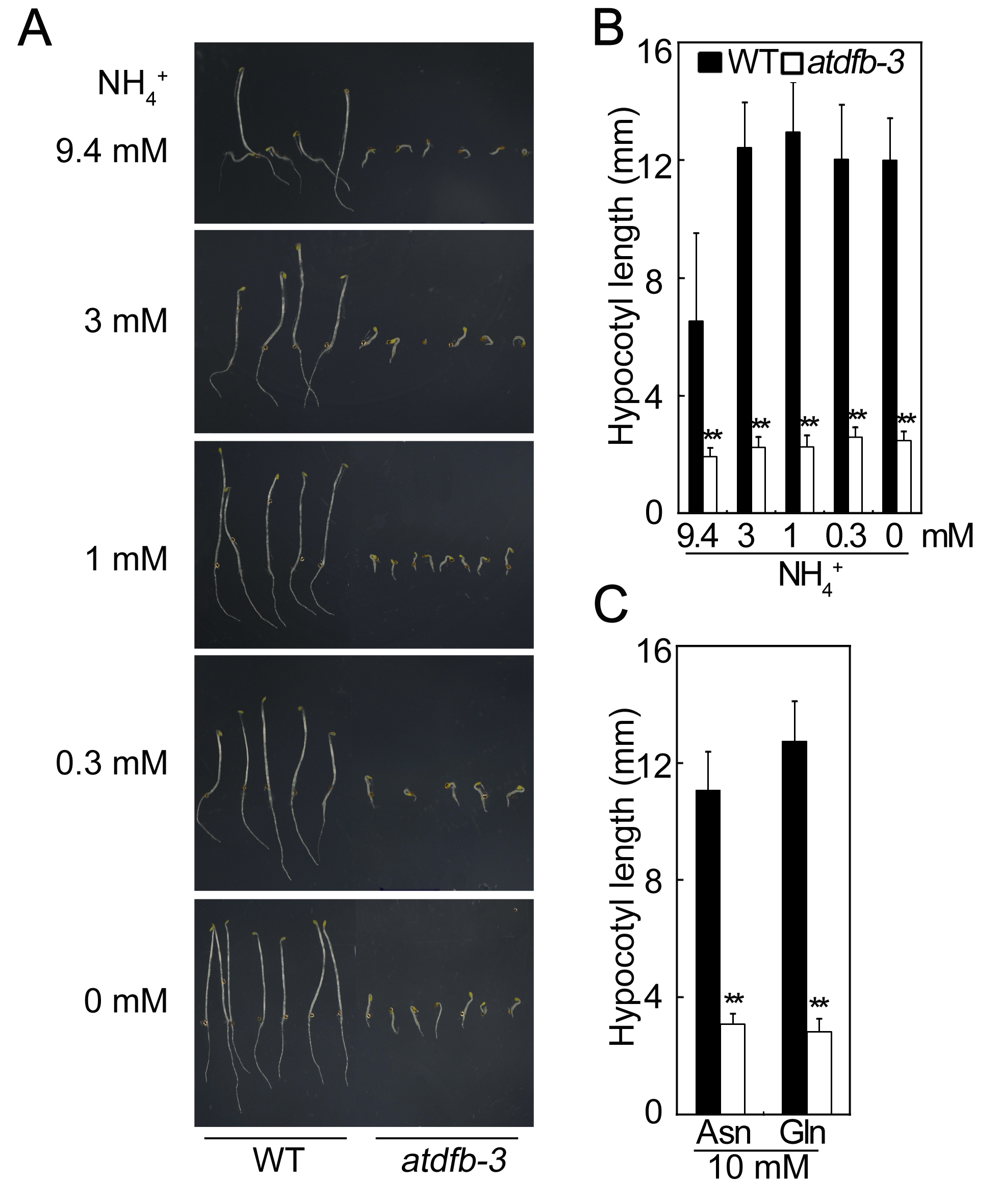


**Figure S2.** Hypocotyl phenotypes of 6-day-old etiolated WT and *atdfb-3* at various concentrations of NH_4_^+^ (A, B) or organic nitrogen (C).


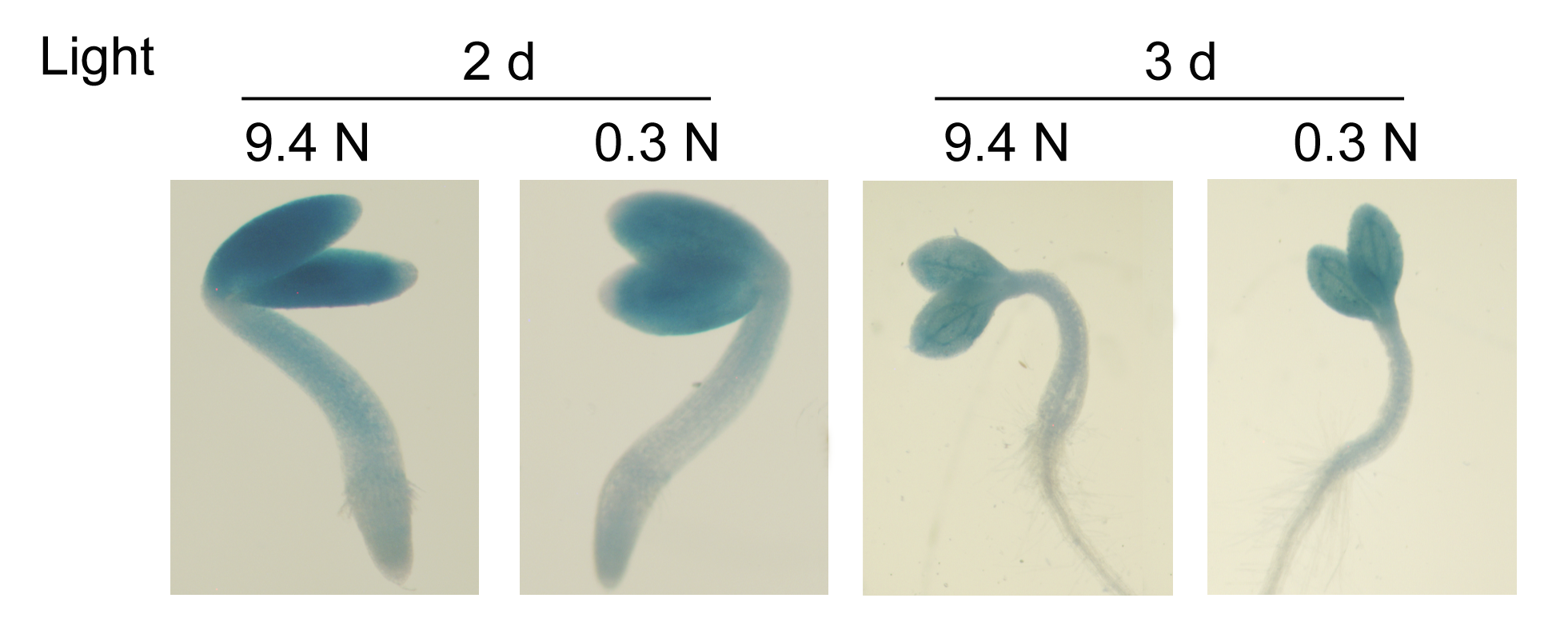


**Figure S3.** GUS staining of 2- and 3-day-old light-grown *AtDFB:GUS* seedlings under 9.4 N or 0.3 N conditions.


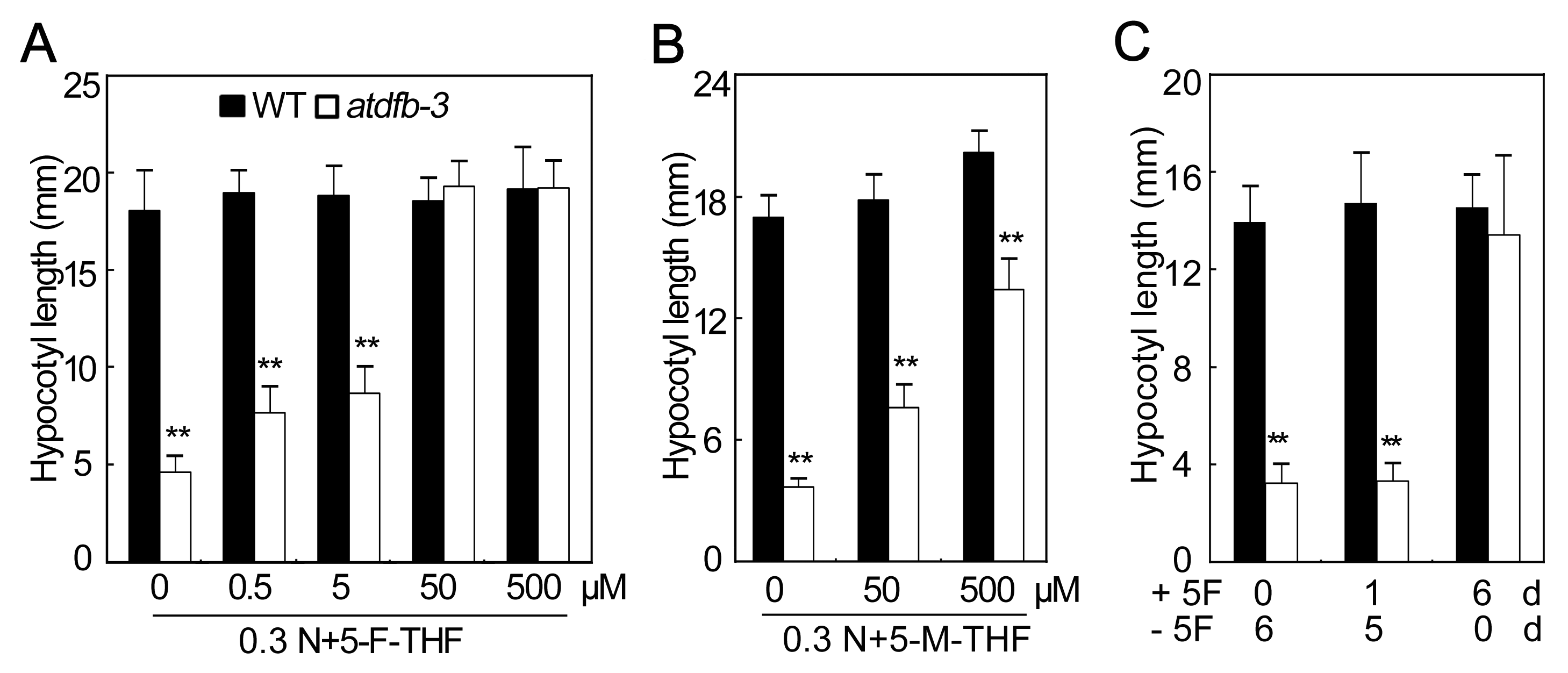


**Figure S4.** Hypocotyl length of WT and *atdfb-3* under N-limited conditions with 5-F-THF or 5-M-THF treatment. **(**A) Hypocotyl length of 7-day-old etiolated WT and *atdfb-3* seedlings after application of various concentrations of 5-F-THF under N-limited conditions*.* (B) Hypocotyl length of 6-day-old etiolated WT and *atdfb-3* seedlings after application of various concentrations of 5-M-THF under N-limited conditions*.* (C) Hypocotyl length of 6-day-old WT and *atdfb-3* etiolated seedlings grown on 0.3 N medium with 50 μM 5-F-THF and then transferred to 0.3 N medium without 5-F-THF for the remaining days.


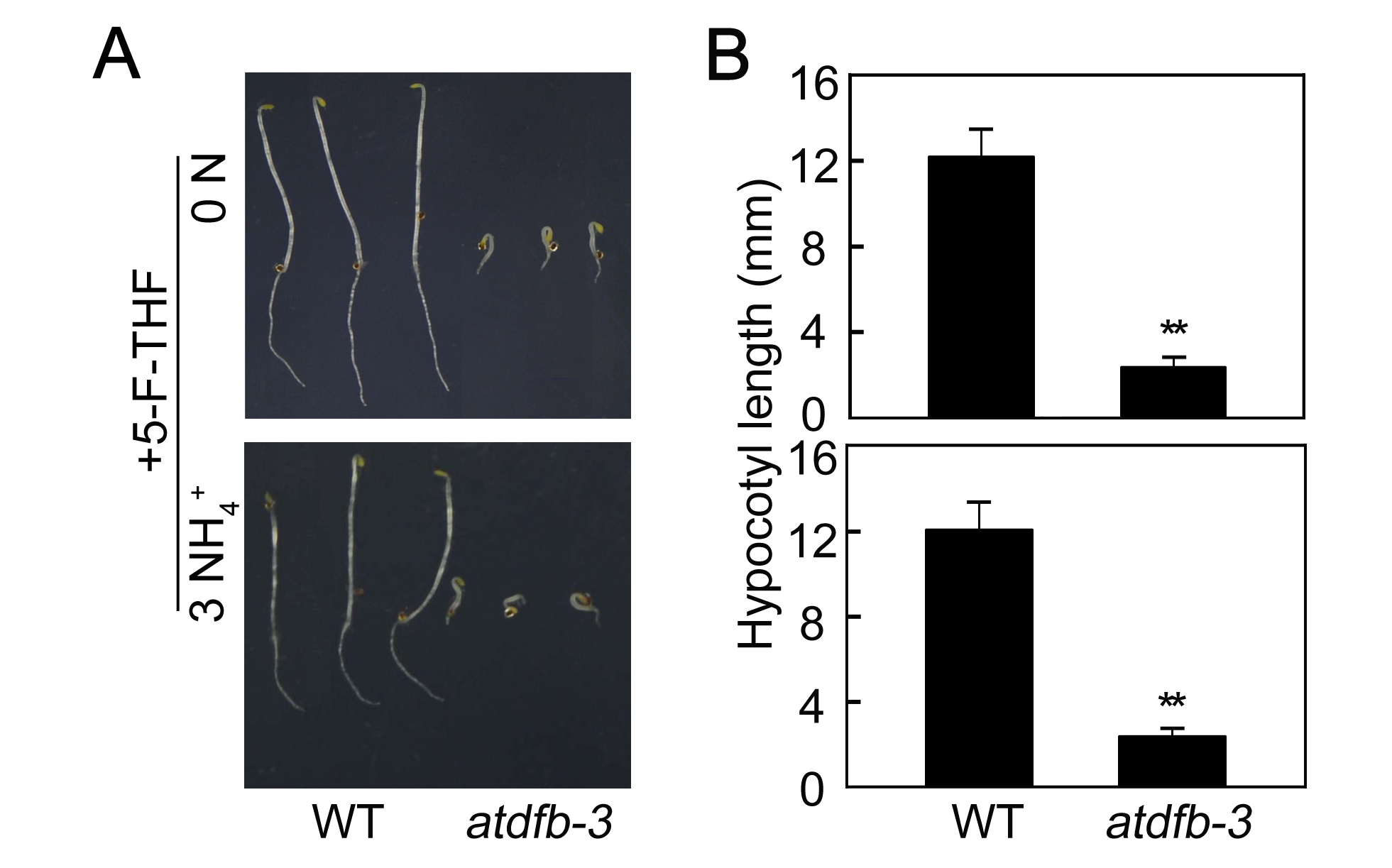


**Figure S5.** Hypocotyl phenotype of 6-day-old etiolated WT and *atdfb-3* under 0 N or NH_4_^+^ with 5-F-THF treatment. (A) Image of hypocotyl phenotype of 6-day-old WT and *atdfb-3* under 0 N or 3mM NH_4_^+^ with 5-F-THF. (B) Hypocotyl length of 6-day-old WT and *atdfb-3* etiolated seedlings grown on 0 N (upper panel) or 3 mM NH_4_^+^ with 50 μM 5-F-THF (lower panel).


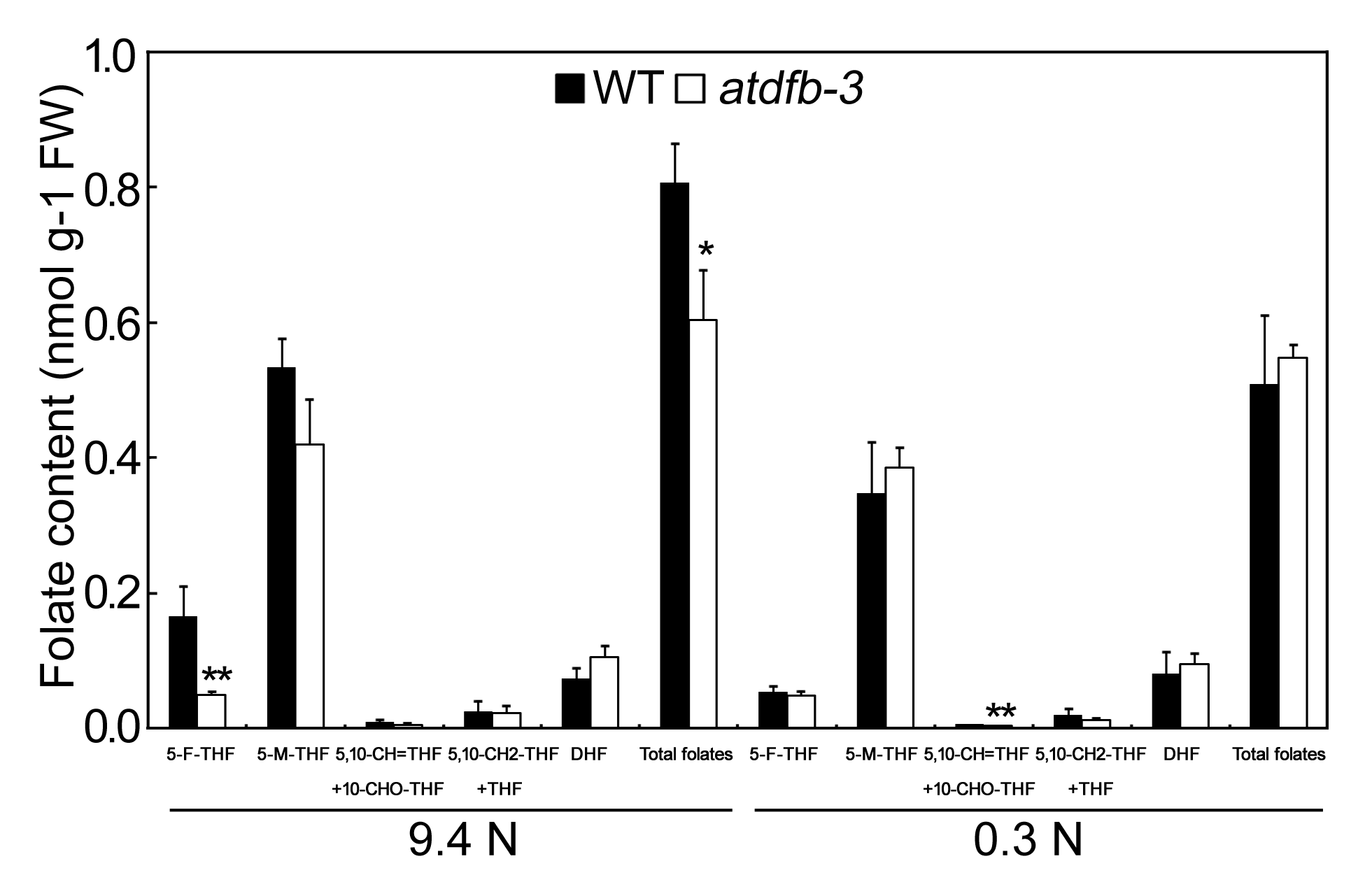


**Figure S6.** Folate profiles in 2-day-old WT and *atdfb-3* germinating seeds under 9.4 N or 0.3 N.


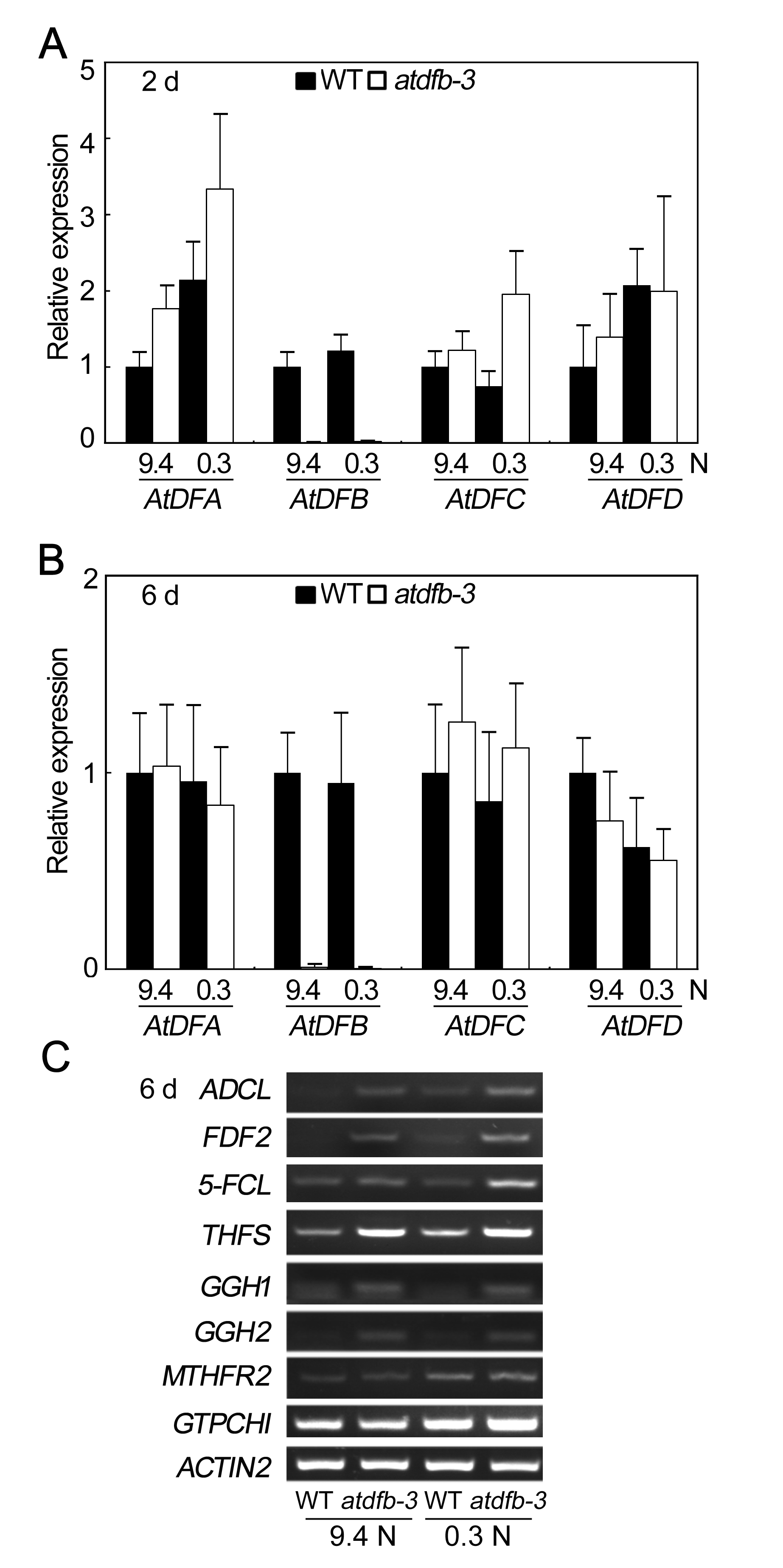


**Figure S7.** Transcript levels of genes involved in folate biosynthesis and metabolism.


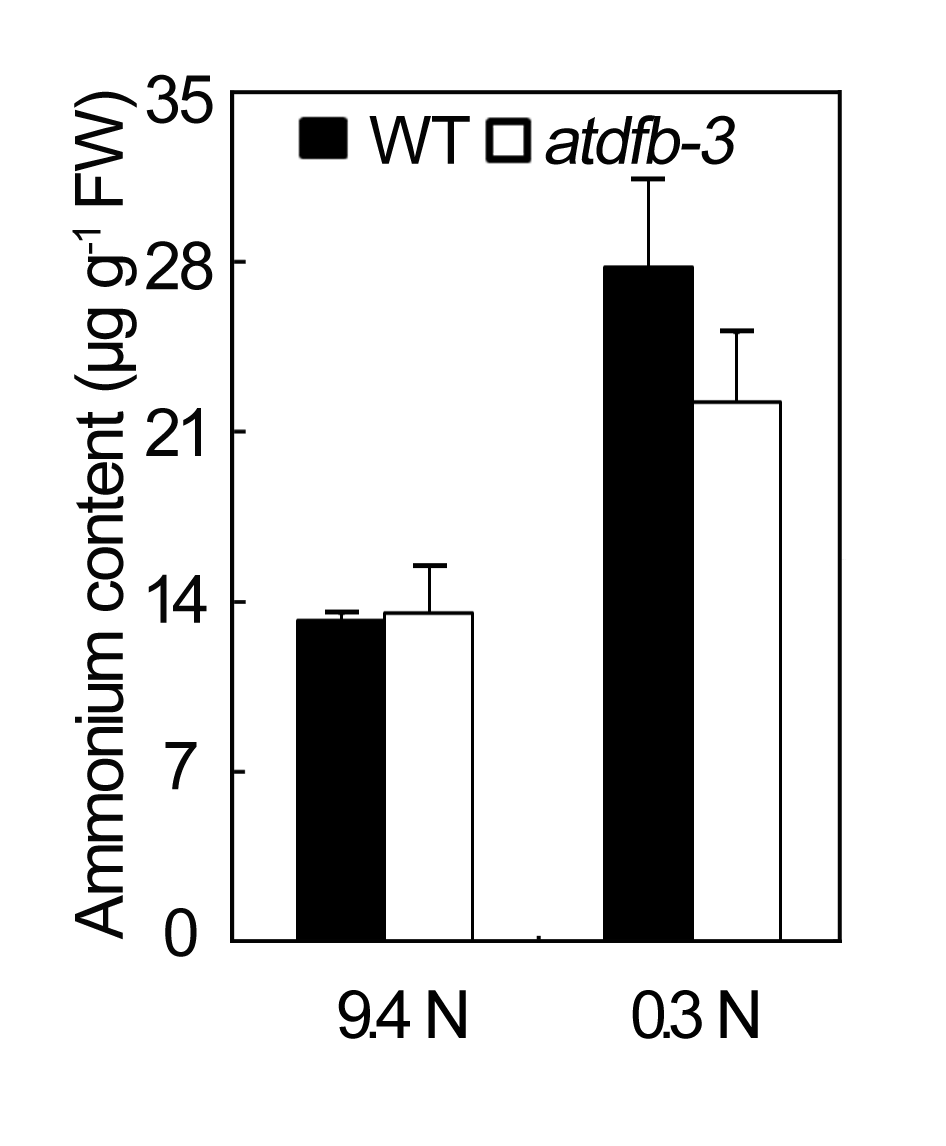


**Figure S8.** Ammonium content in 6-day-old WT and *atdfb-3* seedlings in the dark.

**Table S1.** Profiles of total folates and various folate species in 2-day-old WT and *atdfb-3* germinating seeds grown on 9.4 N or 0.3 N medium in the dark.

Data are means ± SD (n = 5). Each replicate consisted of 100 mg of pooled plant material. Values from one representative experiment are shown. Values in parentheses are the percentages of each folate species relative to the total folates. Values in bold with * indicate a significant difference at P < 0.05, and values with ** indicate a highly significant difference at P < 0.01 (Student’s *t*-test).

|  |  |  | |  | |  |  |  |  |
| --- | --- | --- | --- | --- | --- | --- | --- | --- | --- |
| N status | genotype | Folate content (nmol g^-1^ FW) | | | | | | | |
|  |  | 5-F-THF | 5-M-THF | | 10-CHO-THF+5,10-CH=THF | | THF+5,10-CH_2_-THF | DHF | Total |
| 9.4 N | WT | **0.166±0.045** | 0.533±0.043 | | 0.009±0.004 | | 0.025±0.016 | 0.073±0.016 | **0.807±0.058** |
|  |  | (21) | (66) | | (1) | | (3) | (9) |  |
|  | *atdfb-3* | **0.050±0.006**** | 0.420±0.068 | | 0.005±0.001 | | 0.023±0.010 | 0.106±0.017 | **0.604±0.074*** |
|  |  | (8) | (69) | | (1) | | (4) | (18) |  |
| 0.3 N | WT | 0.054±0.009 | 0.348±0.076 | | **0.006±0.000** | | 0.020±0.010 | 0.081±0.033 | 0.509±0.101 |
|  |  | (11) | (68) | | (1) | | (4) | (16) |  |
|  | *atdfb-3* | 0.049±0.007 | 0.386±0.029 | | **0.004±0.001**** | | 0.013±0.003 | 0.096±0.015 | 0.548±0.019 |
|  |  | (9) | (70) | | (1) | | (2) | (18) |  |
|  |  |  |  | |  | |  |  |  |

**Table S2.** Profiles of total folates and various folate species in 6-day-old WT, *atdfb-3*, and *AtDFB* complemented (COM) etiolated seedlings grown on 9.4 N or 0.3 N medium.

Data are means ± SD (n = 5). Each replicate consisted of 100 mg of pooled plant material. Values from one representative experiment are shown. Values in parentheses are the percentages of each folate species relative to the total folates. Values in bold with * indicate a significant difference at P < 0.05, and values with ** indicate a highly significant difference at P < 0.01 (Student’s *t*-test).

|  |  |  | |  | |  |  |  |  |
| --- | --- | --- | --- | --- | --- | --- | --- | --- | --- |
| N status | genotype | Folate content (nmol g^-1^ FW) | | | | | | | |
|  |  | 5-F-THF | 5-M-THF | | 10-CHO-THF+5,10-CH=THF | | THF+5,10-CH_2_-THF | DHF | Total |
| 9.4 N | WT | **0.10±0.01** | **0.28±0.03** | | 0.01±0.00 | | 0.03±0.01 | **0.07±0.01** | **0.49±0.05** |
|  |  | (21) | (57) | | (2) | | (6) | (14) |  |
|  | *atdfb-3* | **0.07±0.01**** | **0.10±0.01**** | | 0.01±0.00 | | 0.02±0.00 | **0.05±0.01*** | **0.25±0.03**** |
|  |  | (28) | (40) | | (4) | | (8) | (20) |  |
|  | *COM* | 0.06±0.01 | 0.22±0.02 | | 0.01±0.00 | | 0.03±0.00 | 0.03±0.00 | 0.35±0.02 |
|  |  | (17) | (63) | | (3) | | (8.5) | (8.5) |  |
| 0.3 N | WT | **0.05±0.01** | **0.29±0.02** | | 0.01±0.00 | | 0.03±0.00 | **0.05±0.01** | **0.43±0.03** |
|  |  | (12) | (67) | | (2) | | (7) | (12) |  |
|  | *atdfb-3* | **0.08±0.01**** | **0.15±0.01**** | | 0.01±0.00 | | 0.05±0.00 | **0.07±0.01*** | **0.36±0.02**** |
|  |  | (22) | (42) | | (3) | | (14) | (19) |  |
|  | *COM* | 0.06±0.01 | 0.22±0.01 | | 0.01±0.00 | | 0.03±0.00 | 0.06±0.02 | 0.38±0.03 |
|  |  | (16) | (58) | | (2) | | (8) | (16) |  |
|  |  |  |  | |  | |  |  |  |
